# Unique Function of the Bacterial Chromosome Segregation Machinery in Apically Growing *Streptomyces* - Targeting the Chromosome to New Hyphal Tubes and its Anchorage at the Tips

**DOI:** 10.1371/journal.pgen.1006488

**Published:** 2016-12-15

**Authors:** Agnieszka Kois-Ostrowska, Agnieszka Strzałka, Natalia Lipietta, Emma Tilley, Jolanta Zakrzewska-Czerwińska, Paul Herron, Dagmara Jakimowicz

**Affiliations:** 1 Faculty of Biotechnology, University of Wroclaw, Poland; 2 Institute of Pharmacy and Biomedical Sciences, University of Strathclyde, Glasgow, United Kingdom; 3 Institute of Immunology and Experimental Therapy, Wroclaw, Poland; University of Geneva Medical School, SWITZERLAND

## Abstract

The coordination of chromosome segregation with cell growth is fundamental to the proliferation of any organism. In most unicellular bacteria, chromosome segregation is strictly coordinated with cell division and involves ParA that moves the ParB nucleoprotein complexes bi- or unidirectionally toward the cell pole(s). However, the chromosome organization in multiploid, apically extending and branching *Streptomyces* hyphae challenges the known mechanisms of bacterial chromosome segregation. The complex *Streptomyces* life cycle involves two stages: vegetative growth and sporulation. In the latter stage, multiple cell divisions accompanied by chromosome compaction and ParAB assisted segregation turn multigenomic hyphal cell into a chain of unigenomic spores. However, the requirement for active chromosome segregation is unclear in the absence of canonical cell division during vegetative growth except in the process of branch formation. The mechanism by which chromosomes are targeted to new hyphae in streptomycete vegetative growth has remained unknown until now. Here, we address the question of whether active chromosome segregation occurs at this stage. Applied for the first time in *Streptomyces*, labelling of the chromosomal replication initiation region (*oriC*) and time-lapse microscopy, revealed that in vegetative hyphae every copy of the chromosome is complexed with ParB, whereas ParA, through interaction with the apical protein complex (polarisome), tightly anchors only one chromosome at the hyphal tip. The anchor is maintained during replication, when ParA captures one of the daughter *oriC*s. During spore germination and branching, ParA targets one of the multiple chromosomal copies to the new hyphal tip, enabling efficient elongation of hyphal tube. Thus, our studies reveal a novel role for ParAB proteins during hyphal tip establishment and extension.

## Author Summary

To proliferate, cells synchronize growth and division with chromosome segregation. In unicellular bacteria, chromosomes segregate during replication by active movement of nucleoprotein complexes toward the cell pole(s). Here, we asked the question how active chromosome segregation occurs in the absence of cell division, during hyphal growth and branching of the filamentous bacterium, *Streptomyces coelicolor*. We show that in multigenomic *Streptomyces* hyphae, the bacterial segregation machinery anchors a single chromosome at the hyphal tip. Through chromosomal anchorage, segregation proteins facilitate chromosome targeting to the newly formed germ tubes or branches. Thus, being adapted for apical growth, in *Streptomyces* hyphae the bacterial segregation machinery imposes a chromosome distribution that is reminiscent of nuclear distribution in apically growing eukaryotic cells such as filamentous fungi.

## Introduction

Chromosome segregation in unicellular bacteria is strictly coordinated with the cell cycle and chromosomes are segregated during their replication and prior to cell division. However, the spatial chromosome organization in bacteria, determined by the position of the origin of replication (*oriC*) in the cell, differs with respect to the morphology and growth strategy of the organism [1–3]. These differences in chromosome organization are reflected in the specific, cell cycle-tuned mechanism of chromosome segregation [4,5]. In most bacterial species (with the exception of *Escherichia coli* and some γ-proteobacteria) efficient chromosome segregation relies on the activity of two proteins, ParA and ParB [4,6]. By binding *parS* sites clustered around *oriC*, ParB assembles this region of the chromosome into a large nucleoprotein complex. Soon after the initiation of replication, ParB complexes are segregated into specific locations of the cell due to interaction with the ATPase, ParA [6–8]. Species-specific differences in spatial chromosome organization are linked with variations in the ParA and ParB choreography. For instance, during vegetative growth of *Bacillus subtilis*, ParB complexes are segregated bi-directionally to opposite cell poles, while in *Caulobacter crescentus* and in the case of *Vibrio cholerae* chromosome I, only one of the two ParB/*oriC* nucleoprotein complexes is moved toward the opposite pole by the ParA assembly [2]. The interaction of ParA with proteins localized at the cell pole, such as PopZ and TipN in *C*. *crescentus* or HubP in *V*. *cholerae*, translate the cell polarity to asymmetric chromosome segregation [9–11]. The chromosome arrangement and mechanism of segregation remains unexplored in multigenomic bacteria such as the filamentous actinobacteria including *Streptomyces*.

*Streptomyces* differ remarkably from other bacteria with their hyphal growth that is reminiscent of filamentous fungi [12]. Moreover, *Streptomyces* elongated hyphal cells contain multiple copies of linear chromosomes. During colony development, two types of hyphal cells are produced: branching vegetative hyphae that form a dense mycelial network and sporogenic hyphae, that are converted into chains of spores by multiple cell divisions. In contrast to most bacteria that extend along the lateral cell wall, *Streptomyces* as other actinobacteria, grow by cell extension at the poles (tips) [13,14]. The apical synthesis of peptidoglycan in Actinobacteria is linked to the activity of the essential coiled-coil protein, DivIVA that localizes at the cell poles [15–17]. What is unique to *Streptomyces* growth, is the unidirectional cell extension at the hyphal tips. In *Streptomyces*, polar growth is directed by a protein complex localized at the hyphal tip (‘polarisome’ or tip-organizing complex, TIPOC), which includes DivIVA and another coiled-coil protein, Scy [16–18]. Branching is initiated by assembly of the polarisome at a site on the lateral wall distant from the extending tip [14,19]. A similar mechanism of branch formation was observed in the filamentous fungus *Neurospora crassa*, suggesting that it is a feature shared between bacteria and eukaryote [20].

The two stages of *Streptomyces* development, vegetative growth and sporulation, differ with respect to the cellular organization and cell cycle events. During sporulation, multiple, synchronized divisions of elongated sporogenic cells are accompanied by condensation and segregation of numerous chromosomal copies [12]. Our earlier studies showed that the segregation proteins ParA and ParB uniformly distribute chromosomes along the long sporogenic cell at the time of its septation [21,22]. During vegetative growth, multigenomic hyphal cells, named hyphal compartments, do not undergo typical cell division. Widely spaced cross walls delimit, but do not separate, hyphal compartments which remain adjacent in long hyphae [23,24]. Very little is known about chromosome organization in vegetative hyphae. Several copies of the chromosomes remain uncondensed and visibly unseparated when visualized by DNA staining in hyphal compartments. FISH (fluorescence *in situ* hybridization) experiments indicated that the ends of linear chromosomes are spatially close and the chromosomes are unevenly distributed in vegetative hyphae [25]. In addition, replisome labeling demonstrated that chromosomes replicate asynchronously within the compartments and follow the extending tip [27,28]. Localization of segregation proteins in vegetative hyphae is significantly different from their localization in sporulating hyphae. ParB was visualized as multiple, irregularly spaced complexes, with a distinct focus located at a constant distance from the hyphal tip [26]. Meanwhile, ParA in vegetative hyphae, localizes exclusively at the hyphal tips (not along the cell as in sporogenic cells), where it interacts with Scy [29]. Even though the localization of the segregation proteins suggested their engagement in the organization of the apical chromosome, chromosome distribution and the role of ParA and ParB during growth of multigenomic vegetative hyphae remains unknown in the absence of chromosomal locus-specific labelling tools.

To understand how hyphal tip extension and branching are coordinated with distribution and segregation of multiple chromosomal copies during vegetative growth of *S*. *coelicolor*, we took advantage of *oriC* labeling and time-lapse fluorescent microscopy. We show that in multigenomic hyphal cells ParA anchors the single apical chromosome and unidirectionally segregates one of the newly replicated *oriC* regions at the tip. During establishment of the new hyphal tip, ParA-mediated apical *oriC* anchorage targets a chromosome from multigenomic cell to a new branch or a germ tube. Our study reveals a unique mechanism for bacterial chromosome segregation that is adjusted to accommodate hyphal growth and branching.

## Results

### All *oriC*s in multigenomic vegetative hyphae are bound by ParB

To address the question how chromosomes are distributed in *S*. *coelicolor* apically extending and branching vegetative hyphae we constructed a fluorescent reporter-operator system (FROS) to mark chromosomal *oriC* regions.

**Fig 1:**
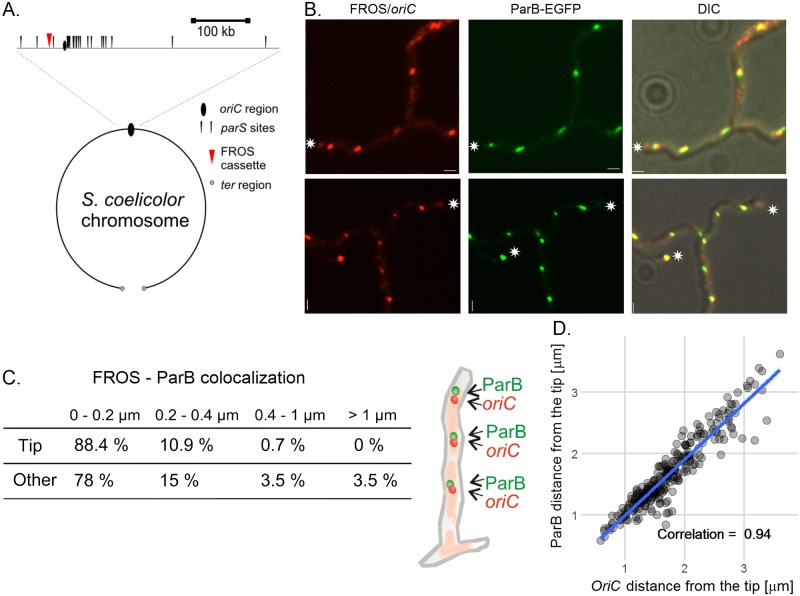
ParB-EGFP complexes co-localize with all *oriC*s in multigenomic *S*. *coelicolor* vegetative hyphae. (A) Scheme of FROS cassette localization in the *S*. *coelicolor* chromosome. (B) Images of ParB-EGFP (green) and FROS (red) foci in vegetative hyphae of FROS *parB-egfp* strain (AK113). The hyphal tips are marked with an asterisk, scale bar—1 μm. (C) Co-localization of FROS and ParB-EGFP foci in AK113 strain along the vegetative hyphae and at the tips of hyphae; the percentage of the foci localizing within the given distance is indicated. (D) Correlation between the distance from ParB-EGFP to the tip of hyphae and distance from FROS signal to tip of FROS *parB-egfp* (AK113) strain hyphae. The scatterplot with a fitted linear model shows data from 16 hyphae measured at 10 minute time intervals. Data were analyzed using a mixed effects model which can compensate for an individual hypha effect and the standard linear model. Results of both models were similar, comparison of Log-likelihoods of both models showed that random effects of individual hyphae were not significant. Correlation was calculated using the Pearson method.

A FROS cassette that contained an array of 120 tandem *tetO* repeats [30] was integrated into the *S*. *coelicolor* chromosome approximately 29 kb from the *oriC* region by *in vitro* transposition and intergenic conjugation [31] (Fig 1A, S1A Fig) resulting in the strain EJTH31. Subsequently the *tetR-mcherry* gene was integrated into the chromosome of this strain on pMS83mCherry resulting in the FROS strain, DJ-NL102 (S1A Fig, strains were verified as shown in S1B and S1C Fig). Analysis of DJ-NL102 hyphae revealed irregularly distributed mCherry foci (the mean distance between foci was 2.0 ± 1.2 μm, S2A and S2B Fig). The foci disappeared upon addition of anhydrotetracycline (aTc) to the culture medium, presumably due to the relief of TetR binding to *tetO* (S2A Fig) [30]. However, in contrast to *E*. *coli* [30], we did not detect any growth impairment or disturbed replication of FROS strain(s) in the absence of aTc (S2C, S2D, S2E and S2F Fig). This suggests that, at least in vegetative hyphae, the binding of TetR-mCherry to the *tetO* array in the FROS cassette did not cause serious replication roadblocks detrimental to growth.

We expected that in hyphal cells ParB-EGFP should co-localize with at least some *oriC*s of the multiple chromosomal copies. To check this, we analyzed a strain AK113 with the FROS cassette and *tetR-mcherry* expressed in a *parB-egfp* background. In AK113 hyphae, 97% of ParB-EGFP foci and mCherry-FROS foci overlapped (distance between foci less than 1 μm) (Fig 1B and 1C). We did not observe any FROS foci unaccompanied by the ParB-EGFP complex at the hyphal tips, suggesting that the tip-proximal chromosome is constantly bound by ParB.

Thus, the FROS labeling of *oriC* regions in *S*. *coelicolor* confirmed irregular distribution of the multiple copies of chromosomes and indicated binding of ParB to each chromosomal *oriC* region in the multigenomic hyphae.

### Only apical *oriC*-ParB complex tightly follows the extending hyphal tip

Earlier studies of chromosome replication in *S*. *coelicolor* vegetative hyphae showed replisome trafficking and suggested that chromosomes follow the extending tip [27]. The constant distance between the FROS/ParB complex and the tip, observed in snapshot analysis, suggests that the tip-proximal chromosome is anchored to the tip during hyphal extension.

**Fig 2:**
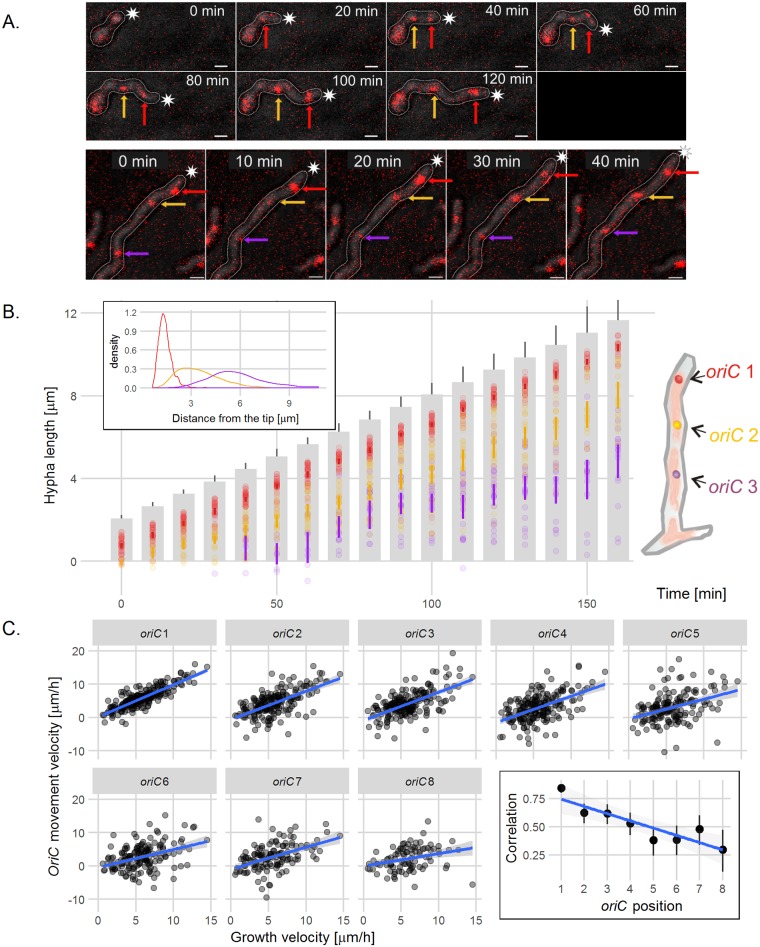
The tip-proximal chromosome follows the extending vegetative hyphae tip. (A) Time-lapse snapshots of the FROS strain (DJ-NL102) germinating spore (top panel) and vegetative hypha (bottom panel). The images are the overlay of TetR-mCherry fluorescence (red) and DIC image (gray) (for separate images of TetR-mCherry fluorescence and DIC see S3D Fig). The arrows indicate: red—*oriC*1 (closest to the tip of the hypha), yellow—*oriC*2, purple—*oriC*3, asterisks indicate the tip of outlined hyphae, scale bar—1 μm. (B) Positions of the FROS complexes in the extending hyphae of FROS strain (DJ-NL102). Grey bars are representations of the extending hyphae with 95% confidence interval for hyphal length and semitransparent colored dots represent *oriC* positions (red–*oriC* 1, yellow–*oriC* 2, purple–*oriC* 3, as shown in the schematic drawing at the right), colored lines indicate 95% mean confidence intervals (analyzed for 41 hyphae). Inset: Distribution (shown as probability density function) of the distances between the hyphal tip and the *oriC* 1 (red), *oriC* 2 (yellow) and *oriC* 3 (purple). (C) Correlation of hyphal extension rate and FROS complex movement calculated for 8 subsequent *oriC*s from the tip. Scatterplots with fitted linear models show data from 20 hyphae measured at 10 minute time intervals, grey area indicates 95% confidence interval for the model. Minus sign means that the distance between the chromosome and the tip is increasing and a plus sign that it is decreasing. Data were analyzed using a mixed effects model, which can compensate for the effect of individual hyphae and a standard linear model. Results of both models were very similar, comparison of Log-likelihoods of both models showed that random effects of individual hyphae were not significant. Inset: the calculated correlation in relation to the *oriC* position in the hyphae with a fitted linear model. Correlations were calculated using Pearson method with 95% confidence intervals.

The application of time-lapse microscopy and the FROS strains (DJ-NL102 and AK113) allowed us to examine the chromosome distribution during hyphal growth (Fig 2, S3A, S3B, S3C and S3D Fig). The earlier observations showed that replication starts before spore germination [27,28], and the germinating spores showed multiple FROS foci, as expected. In spore germ tubes (Fig 2A top panel, S3A and S3D Fig top panel, S1 Movie) and extending vegetative hyphae of 24 hours old colonies (Fig 2A bottom panel, S3D Fig, bottom panel), the distance between the hyphal tip and the first, tip-proximal FROS complex was constant (1.4 ± 0.4 μm), demonstrating that the first *oriC* follows the elongating tip (Fig 2B). Interestingly, the distance between the tip and the FROS complexes located further from the tip was more variable during hyphal extension (Fig 2B, inset). We compared the tip-proximal (*oriC* 1) and tip-distal complexes (*oriC* 2—*oriC* 8) by analyzing the correlation between their movement and tip growth rates (Fig 2C). The correlation was high for the first chromosome and decreased with an increasing distance between the *oriC*s and the extending tip.

Next, we measured the distance between the hyphal tip and the first FROS signal in extending hyphae of FROS, *parB-egfp* strain (AK113) and plotted it against the distance between the ParB complex and the tip (Fig 1D). This analysis confirmed that the positions of the ParB and FROS signals at the hyphal tip were highly correlated and average distance between both complexes and the tip was 1.5–2.0 μm.

Thus, the time-lapse analysis confirmed that chromosomes follow the extending hyphal tip. Markedly, only the first *oriC* remains tightly associated with the extending hyphal tip and maintains a constant distance to it.

### ParAB proteins localize *oriC* at a constant distance to the tip

ParB binds the *oriC*s of each chromosome along the hyphae, but only the first one maintains a constant distance and follows the extending hyphal tip. As ParA in vegetative hyphae is localized at the tip [21, 29](S4 Fig), we hypothesized that the presence of ParA and its interaction with the tip-proximal ParB complex are critical for the localization of the *oriC*/ParB complex at the constant distance from the tip. Analysis of FROS-marked *oriC* regions in the Δ*parB* and Δ*parA* background allowed us to verify this.

**Fig 3:**
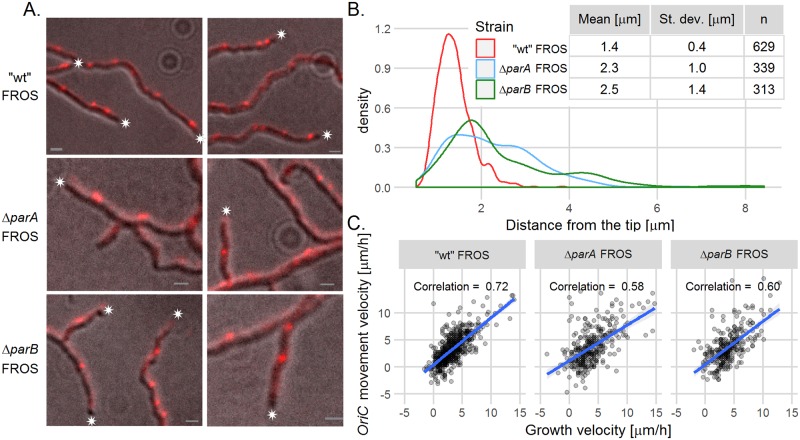
The constant distance between *oriC* and the hyphal tip is dependent on ParAB. (A) Images of FROS in the “wild type” FROS strain (DJ-NL102), Δ*parA* FROS (AK115) and Δ*parB* FROS (AK114) strains. The images are the overlay of TetR-mCherry fluorescence (red) and DIC image (grey), asterisks indicate the tip of hyphae, scale bar—1 μm. (B) Distribution (shown as probability density function) of the distances between the hyphal tip and tip-proximal FROS signal in “wild type” FROS (DJ-NL102), Δ*parA* FROS (AK115) and Δ*parB* FROS (AK114) strains. (C) Correlation between hyphal extension rate and the tip-proximal *oriC* movement velocity in “wild type” FROS (DJ-NL102), Δ*parA* FROS (AK115) and Δ*parB* FROS (AK114) strains (analyzed for 41 of DJ-NL102, 31 AK115 and 30 AK114 hyphae). Scatterplots with fitted linear models, grey area indicates 95% confidence interval for the model.

The snapshots analysis revealed that in the Δ*parB* and Δ*parA* (AK114 and AK115) mutant strains, the tip-proximal FROS complex was further away from the hyphal tip than in the wild type FROS strain. The distances between tip-proximal FROS complexes and the tip were 2.5 ± 1.4 μm in Δ*parB*, 2.3 ± 1.0 μm in Δ*parA* and 1.4 ± 0.4 μm in “wild type” strain (Fig 3A and 3B, differences between mutants and the wild type strain were statistically significant, p<0.001, verified with ANOVA and a *post hoc* Games-Howell test). Moreover, the position of the apical FROS complex exhibited higher variation in Δ*parB* and Δ*parA* strains than in the wild type strain (demonstrated with an F-test; the ratio of variances was 6.3 ± 1.2 for Δ*parA* and 11.9 ± 2.3 for Δ*parB* in relation to the wild type strain (p<2.2e-16)). We also observed that in the Δ*parA* strain, but surprisingly not in the Δ*parB* strain, the distance between the edge of nucleoid and the hyphal tip was increased, when compared to the wild type FROS strain (S5 Fig, only the difference between the Δ*parA* and the wild type was statistically significant, p<0.001 verified with ANOVA and *post hoc* Tukey’s test).

The increased distance of the tip-proximal *oriC* to the extending hyphal tip in the Δ*parA* (AK115) and Δ*parB* (AK114) strains was confirmed by time-lapse analysis of FROS complex dynamics (S6 Fig). Plotting the correlation between the hyphal extension rate and *oriC* movement showed that the association of the first *oriC* with the hyphal tip was visibly decreased in the mutant strains (Fig 3C). Although chromosome trafficking in the hyphae of the Δ*parA* and Δ*parB* strains was maintained, we noted a slightly increased variation of the distances between *oriC* 2 or *oriC* 3 and the tip (S6 Fig).

Our analyses show that in the Δ*parA* and Δ*parB* mutant strains, chromosome trafficking in hyphae was maintained albeit slightly disturbed. Moreover, both segregation proteins ParA and ParB were essential for anchorage of the tip-proximal chromosome *oriC* region at the tip of the extending hyphae.

### ParA positions the tip-proximal ParB–*oriC* complex

Having established that both segregation proteins are required for the tip-proximal *oriC* localization, we sought confirmation that ParA anchors and organizes the first ParB complex. To address this question, we compared the localization of the ParB complex in a set of strains with different *parA* modifications: Δ*parA* (J3318), a *parA* overexpression strain (DJ532) and a strain with a mutation that abolishes the interaction with ParB and Scy—*parA*_*mut*_ (DJ598) [29].

**Fig 4:**
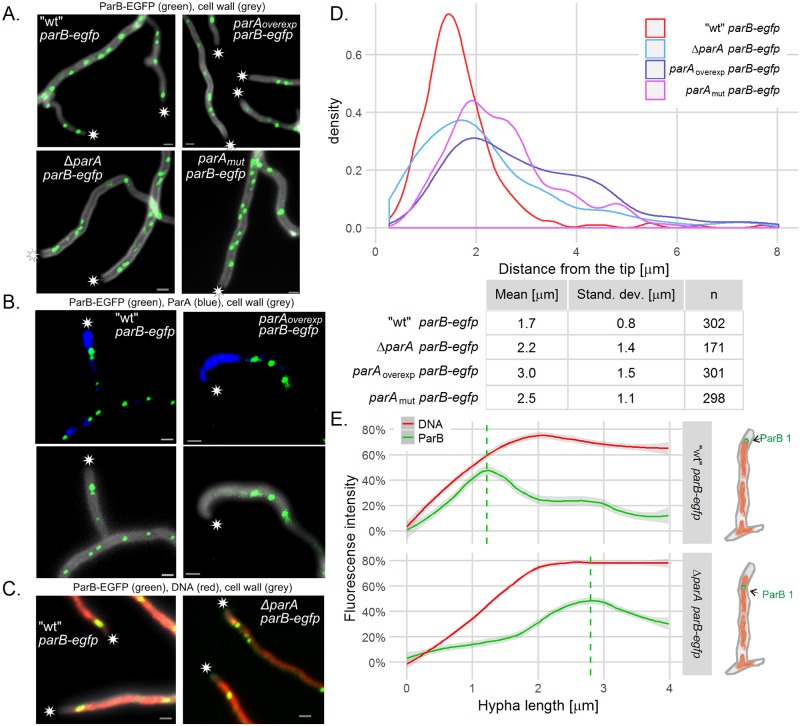
Localization of ParB complex is dependent on ParA. (A) Images of ParB-EGFP (green) complexes in the hyphae of”wild type” *parB-egfp* (J3310) Δ*parA parB-egfp* (J3318), *parA*_*overexp*_ *parB-egfp* (DJ532), *parA*_*mut*_ *parB-egfp* (DJ598) strains, merged with cell wall staining (gray). (B) Co-localization of ParB-EGFP (green) with immunostained ParA (blue) in “wild type” *parB-egfp* (J3310) and *parA*_overexp_ *parB-egfp* (DJ532). Top panel shows the ParA immunofluorescence (blue) merged with ParB-EGFP fluorescence (green). The bottom panel shows ParB-EGFP fluorescence (green) merged cell wall staining (grey). (C) Localization of ParB-EGFP (green) within the nucleoid (DNA staining—red) in”wild type” *parB-egfp* (J3310) and in Δ*parA parB-egfp* (J3318). In panels A, B and C asterisks indicate the tip of hyphae and scale bars—1 μm. (D) The distribution (shown as probability density function) of the distances between the hyphal tip and the tip-proximal ParB-EGFP complex in „wild type” *parB-egfp* (J3310), Δ*parA parB-egfp* (J3318), *parA*_*overexp*_ *parB-egfp* (DJ532), *parA*_*mut*_ *parB-egfp* (DJ598) (analyzed for 170–300 hyphae). (E) Fluorescence intensity of ParB-EGFP and DNA stain measured from the hyphal tip in “wild type” *parB-egfp* (J3310) and Δ*parA parB-egfp* (J3318) (15 and 21 hyphae analyzed). For each hypha, the fluorescence signal was normalized so that the maximum signal was 100%. Lines are models fitted using a Loess algorithm implemented in the R program, grey area indicates 95% confidence interval. Dashed line shows maximum ParB fluorescence intensity as calculated by the model.

In *parA* mutant strains, the tip-proximal ParB complex was delocalized and positioned further away from the tip than in the wild type strain. The distance between the ParB-EGFP complex and the tip was 2.2 ± 1.4 μm in the Δ*parA* strain, 3.0 ± 1.5 μm in the strain overexpressing *parA* and 2.5 ± 1.1 μm in the *parA*_*mut*_ strain in comparison to 1.7 ± 0.8 μm in the control *parB–egfp* strain (Fig 4A and 4D, the differences between the mutants and the wild type strain were statistically significant, p<0.001, calculated with ANOVA and *post hoc* Games-Howell test). The measured distance of ParB-EGFP complex to the tip somewhat differs from earlier reports [26] and from *oriC*–tip distance (1.4 ± 0.4 μm, see above). This is possibly due to different sample processing and data analysis (use of cell wall staining instead of transmitted light images (Figs 1, 2 and 3) presumably affects the analysis of the hyphal tip position). The variation of the tip-proximal ParB complex positioning in the *parA* mutant strains was very high (shown with an F-test, the ratio of variances 3.0 ± 0.8 for Δ*parA*, 3.2 ± 0.7 for *parA* overproduction and 1.7 ± 0.4 for *parA*_*mut*_ strain (p < 0.001)) (Fig 4D).

Overexpression of *parA* resulted in the most notable change of the ParB complex position. Immunostaining of ParA in the wild type *parB-egfp* strain revealed that the ParB complex was positioned at the edge of the ParA signal (Fig 4B). The overproduction of ParA led to a huge accumulation of ParA at the hyphal tips and shifted the ParB complex away from the tip.

We showed before that tip localization of ParA is dependent on Scy and ParA_mut_ protein is mislocalized [29]. However, since the ParA_mut_ protein does not interact with both, ParB and Scy, on the basis of *parA*_*mut*_ strain analysis, we cannot conclude that the Scy dependent localization of ParA determines localization of ParB complex. The analysis of ParB-EGFP foci in the Δ*scy*, *parB-egfp* strain (BD05) showed that in the absence of Scy, the distance between the tip and the apical ParB-EGFP focus in the newly formed branches was much more varied than in the “wild type” strain J3310 (S7 Fig). This reinforced the notion that Scy-ParA interaction is required to establish anchorage for *oriC*/ParB complex soon after branch emergence.

Finally, we examined whether ParA influences the overall organization of the tip chromosome through the ParB complex and *oriC* anchorage. To address this question, we analyzed the localization of ParB complexes in conjunction with DNA staining (Fig 4C). In the wild type *parB-egfp* strain, the ParB complex was found at the tip-proximal edge of the stained nucleoid, whereas in the Δ*parA* strain, the ParB complex was located further away from the tip than the edge of the nucleoid. Measurement and plotting of the ParB fluorescence and DNA staining intensities confirmed that in the absence of ParA, the tip-proximal orientation of the *oriC*/ParB complex at the edge of the nucleoid was lost (Fig 4E).

To recapitulate, analyses of the *parA* mutant strains revealed that the distance between the first ParB complex and the tip is dependent on ParA. ParA interactions with Scy and ParB are presumably required to establish the chromosomal tip anchorage. Furthermore, the ParB complex is positioned at the edge of the tip ParA assembly, and the interaction between the ParB complex and ParA orientates the nucleoid with the *oriC* region toward the tip of hyphae.

### After replication ParA anchors at the tip one of the newly–replicated *oriC*

We have demonstrated that ParA, presumably through interaction with Scy, anchors the *oriC*/ParB complex at the tip, assuring that it follows the extending hyphal tip. This raises the question how this anchorage is established after *oriC* duplication. To answer this question, we used a strain with the EGFP tagged replisomes (DnaN-EGFP, J3337, [28]) as the parent strain for the introduction of the FROS system, to monitor duplication of the tip proximal *oriC* by time-lapse analysis.

**Fig 5:**
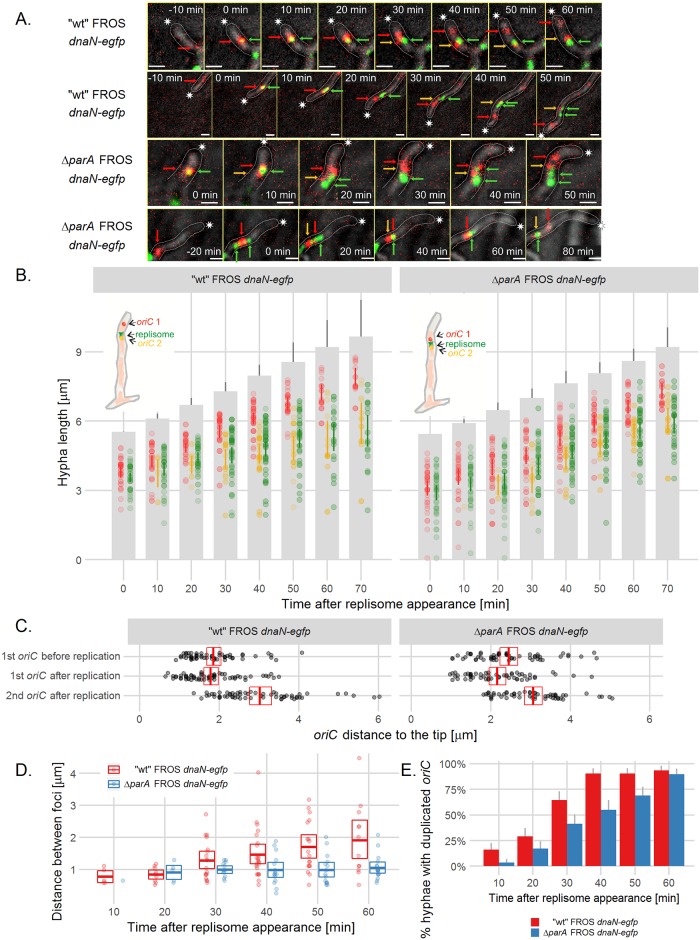
*oriC* is captured at the tip soon after replication. (A) Time-lapse snapshots of FROS (TetR-mCherry fluorescence, red) and DnaN-EGFP foci (green) in the extending hyphae of “wild type” FROS *dnaN-egfp* (AK122) (top panel) and Δ*parA* FROS *dnaN-egfp* (AK123) (bottom panel) strains. The fluorescence images are merged with the DIC images (grey) (for separate images of TetR-mCherry overlaid with DnaN-EGFP fluorescence and DIC see S8 Fig). Asterisks indicate the tip of the outlined hyphae, green arrows point to the replisome complex, red arrows point to the tip-proximal *oriC*, yellow arrows point to tip-distal *oriC*, scale bar—1 μm. (B) Position of the *oriC* and DnaN-EGFP complexes in relation to the tips of extending hyphae in “wild type” FROS *dnaN-egfp* (AK122) (left panel) and Δ*parA* FROS *dnaN-egfp* (AK123) (right panel) strains (analyzed for 32 AK122 hyphae and 29 AK123 hyphae). Grey bars are representations of the extending hyphae with 95% confidence interval for hyphae length and semitransparent colored dots represent *oriC* positions (red: tip-proximal *oriC* 1, yellow: tip-distal *oriC* 2, green: replisome, as shown on the schematic drawings), colored lines indicate 95% mean confidence intervals. (C) The distance between the tip and the tip-proximal *oriC* 10–20 minutes before and 10–20 minutes after *oriC* duplication in “wild type” FROS *dnaN-egfp* (AK122) and Δ*parA* FROS *dnaN-egfp* (AK123) strains. (D) Distance between duplicated *oriC*s at the indicated time after replisome appearance in “wild type” FROS *dnaN-egfp* (AK122) and Δ*parA* FROS *dnaN-egfp* (AK123) strains. In C and D panel crossbars show the mean with 95% confidence intervals. (E) Percentage of hyphae in which the duplicated *oriCs* could be detected at the indicated time after replisome appearance. Error bars show 95% confidence intervals.

We set out to examine how daughter *oriC*s become anchored after replication. Possible scenarios included a loss of connection with the tip during *oriC* replication; a close association of both newly replicated *oriC*s with the tip, followed by a loss of anchorage of one of the duplicated *oriC*s; or anchorage of only one of the newly replicated *oriC*s to the tip. By analyzing the time-lapse images of the FROS strain expressing *dnaN-egfp* (strain AK122), we observed the appearance of a DnaN focus close to a tip-proximal FROS focus (Fig 5A top panel, B, S8 Fig, top panel, S2 Movie). 10 min after the replisome appearance, we detected, both newly replicated *oriCs* separated by a distance of around 0.8 μm in almost 15% of the hyphae. Twenty minutes later, duplicated *oriC*s separated by a distance of roughly 1.3 μm were visible in approximately 70% of hyphae (Fig 5B, 5D and 5E). However, the distance between the tip and tip-proximal *oriC* was the same 20–10 minutes before and 10–20 minutes after *oriC* duplication (Fig 5C). Thus, after duplication, the tip-proximal daughter *oriC* did not move toward the tip, but maintained a constant distance to the tip (Fig 5B and 5C). Interestingly, the second of the newly replicated *oriCs* remained co-localized with the replisome in the subsequent time-lapse images (Fig 5A top panel, Fig 5B, S8 Fig, top panel). The distance between the tip and tip-distal daughter *oriC*s increased as hyphae extended (Fig 5B and 5C). These results indicate that while tip the proximal *oriC* follows the tip, the second *oriC* stays behind the extending tip.

In the Δ*parA* FROS *dnaN-egfp* strain (AK123), both daughter *oriC*s remained closely associated after duplication (Fig 5A bottom panel, S8 Fig, bottom panel, S3 Movie). In the Δ*parA* strain (AK123), we detected double FROS foci later after replisome appearance than observed in the “wild type” AK122. Daughter *oriC*s separated by a distance of approximately 0.9 μm were visible 20 minutes after replisome appearance in only 12% of the hyphae (Fig 5A bottom panel, Fig 5B right panel, D, E). Approximately 30 minutes after replisome detection, the average distance between the duplicated *oriCs* reached 1 μm in about 35% of hyphae, but it did not exceed this value (for comparison, in the wild type hyphae at this time point the distance was 1.3 μm in 70% of hyphae). Interestingly, in the Δ*parA* strain, both *oriCs* and replisomes still followed the extending hyphal tip, but the distance between the first *oriC* and the hyphal tip remained greater than that found for the “wild type” AK122 strain (Fig 5C). This observation indicates that, although both newly duplicated *oriCs* still move behind the extending tip independently of ParA (in Δ*parA* strain), their separation is less efficient and the first *oriC* is not attached to the hyphal tip.

Since ParB complexes form at each *oriC* in the multigenomic hyphal compartment we expected that shortly after replication, both daughter *oriC*s should be bound by ParB. To test this we used the strain expressing *parB-egfp* and *dnaN-mcherry* (AK101). Time-lapse analysis showed that ParB complex duplication, as *oriC* duplication, was detected 30 min after replisome appearance at the tip in almost 70% of hyphae (S9 Fig).

In order to confirm that segregation of duplicated *oriC* in hyphae is efficient in the presence of ParA we also checked how the newly replicated tip-distal *oriC*s are separated in the hyphal stem. The analysis of the distances between FROS foci after their duplication showed that the separation of tip-distal foci is much less efficient than separation of tip-proximal foci which occurs in presence of ParA (S10 Fig).

In summary, during replication the position of the tip-proximal daughter *oriC* does not change in relation to the tip, while the other *oriC* co-localizes with the replisome and gradually falls behind the extending tip. The tip-distal *oriC* are not separated as efficiently after duplication as tip-proximal foci. In the Δ*parA* strain, the tip anchorage of *oriC* is impaired and separation of daughter tip-proximal *oriCs* is inefficient. Thus, although both daughter *oriC*s are bound by ParB shortly after their duplication, immediately after replication ParA captures one of the apical ParB/*oriC* complexes and maintains its constant distance to the tip.

### The ParA targets the chromosome to the new hyphal tips

The elimination of ParA affects chromosome segregation during sporulation, but it has not been reported that ParA is required for vegetative growth. As our experiments indicated that ParA anchors the *oriC*/ParB complex of the tip-proximal chromosome in the vegetative hyphal tips, we expected that its elimination should affect the population of the new hyphal tips with the chromosomes. Thus, using time-lapse microscopy analysis, we re-investigated the influence of ParA and ParB on vegetative growth with a particular focus on spore germination and branching.

Microscopy analysis of spore germination showed that a *parA* deletion decreased the efficiency of germ tube formation. The number of germinating spores during the 16-h time-lapse microscopy observation dropped from approximately 61% in the “wild type” FROS strain (DJ-NL102) to approximately 32% in the Δ*parA* strain (AK115). Interestingly, the spores of the Δ*parB* strain (AK114) germinated with a similar efficiency (58%) as the “wild type” strain.

**Fig 6:**
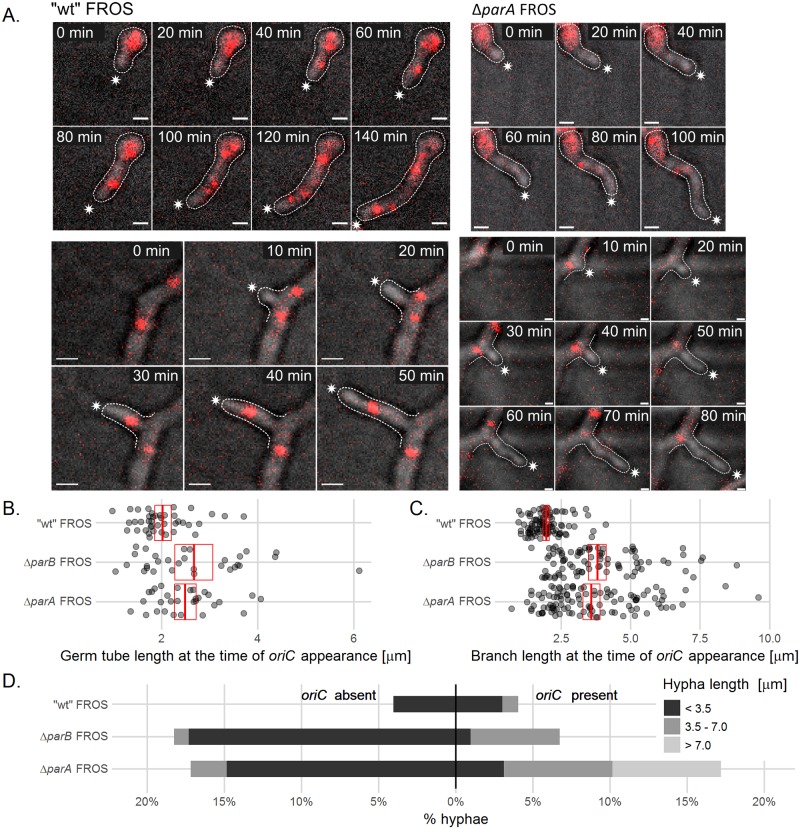
ParA anchorage is required for chromosome migration to germ tubes and hyphal branches. (A) Time-lapse snapshots of FROS complexes during germination (top panels) and branch formation (bottom panels) in “wild type” FROS (DJ-NL102) and Δ*parA* FROS (AK115) strains. The images are the overlay of TetR-mCherry (red) fluorescence and DIC image (for separate images of TetR-mCherry fluorescence and DIC image see S11 Fig). The asterisks indicate the tip of outlined hyphae, scale bar—1 μm. (B) Germ tube length at the time of the first *oriC* appearance in “wild type” FROS (DJ-NL102), Δ*parA* FROS (AK115) and Δ*parB* FROS (AK114) strains (analyzed for 41 DJ-NL102, 31 AK115 and 30 AK114 germ tubes). (C) Branch length at the time of the first *oriC* appearance in “wild type” FROS (DJ-NL102), Δ*parA* FROS (AK115) and Δ*parB* FROS (AK114) strains. 95 hyphae of DJ-NL102, 106 of AK115 and 85 of AK114 strain were analyzed. In B and C, red crossbars show means with 95% confidence intervals. (D) Percentage and length of stalled branches with and without the FROS signal in “wild type” FROS (DJ-NL102), Δ*parA* FROS (AK115) and Δ*parB* FROS (AK114) strains. Hyphae were classified as stalled if no re-initiation of growth could be observed until the end of the experiment or for at least one hour (whichever was longer). 99 hyphal branches were analyzed of the DJ-NL102, 128 of AK115 and 104 of AK114 strain.

According to our hypothesis, the germination of the Δ*parA* strain may be impaired due to the less efficient population of the germ tube by the chromosomes. To test this hypothesis, we used the FROS strains (control strain DJ-NL102, Δ*parA*-AK115 and Δ*parB*-AK114) to analyze the influence of *parA* and *parB* deletion on the length of the germ tube at the time of the *oriC* signal appearance (Fig 6A top panel, S11 Fig, top panel). We found that in the germinating spores of the wild type FROS strain (DJ-NL102), the FROS signal was detected when the germ tube reached the 2 μm length, but in Δ*parA* and Δ*parB* strains, the germ tubes were longer (2.5 μm and 2.7 μm, respectively) at the time when the FROS signal appeared (Fig 6B, the differences were statistically significant p<0.01, verified by ANOVA and *post hoc* Games-Howells test). This suggests that elimination of ParA and ParB delayed chromosome migration from the spore to the elongating germ tube.

The chromosome anchorage should not only be important during formation of the germ tube but also during new branch formation. Indeed, the time-lapse experiment and analysis of the length of emerging new branches at the time of FROS signal appearance showed that, while the wild type FROS signal could be detected in hyphal branches when they were 2 μm on average (similarly to germ tubes). In Δ*parA* and Δ*parB* strains (AK115 and AK114) the hyphae were longer than in wild type at the time of FROS signal detection, approximately 3.6 μm and 3.8 μm long, respectively, (Fig 6A, bottom panel and Fig 6C, S11 Fig, bottom panel, S4 and S5 Movies). This suggests that ParA and ParB elimination impairs also the migration of chromosomes from the hyphal stem to the newly formed branch.

Interestingly, we noted that chromosome targeting to the new hyphal branch does not need to follow *oriC* duplication in hyphal stem. In 60% of observed new hyphal branches, we could not detect *oriC* duplication during 30 minutes proceeding *oriC* targeting to hyphal branch (Fig 6A). Thus, targeting of the chromosome to new hyphal branches is not accompanying the chromosome replication.

The impaired chromosome population of new branches might also affect their extension. Thus, we investigated the extension of the new branches in “wild type” (DJ-NL102), Δ*parA* (AK115) and Δ*parB* (AK114) FROS strains. In the “wild type” strain, only a small fraction of branches (8%) stopped extension, mostly before they reached a length of 3.5 μm (Fig 6D). Interestingly, in the Δ*parA* and Δ*parB* strains, the percentages of stalled short branches were markedly increased in comparison to the wild type strain. In both strains, approximately 18% of the branches stopped extension when they were still shorter than 3.5 μm, whereas some were stalled when their length was within the range between 3.5 μm and 7.0 μm (7% in Δ*parA* and 5.8% in in Δ*parB*, in contrast to 1% in wild type) or even longer than 7 μm (7% in Δ*parA*, in contrast to 0% in wild type and Δ*parB*). Most of the stalled branches did not show the FROS signal; however, some of the branches that stopped growth contained chromosomes (4% in wild type, 17.1% Δ*parA* and 6.8% in Δ*parB*), as judged by the detection of the FROS signal. This observation indicated that elimination of either ParA or ParB affects the extension of short branches, presumably through their impaired population with chromosomes. Interestingly, the lack of ParA (but not ParB) also disturbs the extension of branches that received chromosomes, possibly due to the lack of chromosome tip anchorage.

In summary, the analyses of germination and branching revealed that ParA tip anchorage of the chromosome is crucial for efficient formation and extension of germ tubes and new branches. Our data indicate that ParA interacting with the ParB complex at one of chromosomal copies directs it to populate the new hyphal tip.

## Discussion

In apically extending and branching *Streptomyces* vegetative hyphae that do not undergo canonical bacterial cell division, multiple copies of the chromosome replicate asynchronously and remain visibly unseparated [24,28]. Due to this mode of growth and a lack of chromosome-locus specific labeling tools available for *Streptomyces* until now, the distribution of nucleoids in vegetative hyphae remained elusive. Furthermore, little was known about the function of segregation proteins during vegetative growth. In contrast to sporulating hyphae, in which ParA extends along the whole cell and accompanies the array or regularly spaced ParB complexes, in vegetative hyphae ParA is exclusively associated with the hyphal tip [21,29]. We noted before the presence of irregularly spaced ParB complexes of varying intensity along the hyphae and the tip-proximal ParB complexes which exhibit the highest fluorescence intensity [26]. That observation is in agreement with the finding that ParA is required for efficient assembly of the ParB nucleoprotein complex [22]. Therefore, we hypothesized that the apical ParB complex assembled in the presence of ParA assures organization of the tip-proximal chromosome during apical extension.

*OriC* labeling using the FROS system revealed that chromosomes follow the extending hyphal tip. The distance between the tip and *oriC* of the apical chromosome is constant. Immediately after *oriC* duplication, ParB complexes are reestablished at both daughter *oriC*s, but only one of them is captured by an apically localized ParA complex to renew the tip anchorage of the apical chromosome. A distinctive feature of *Streptomyces* tip-proximal replication is that one of the daughter *oriC*s is abandoned by the segregation machinery. We suggest that the presence of ParA, exclusively at the tip, most likely explains segregation and anchoring of the tip-proximal *oriC* alone. Thus, at the tips of *Streptomyces* vegetative hyphae, *oriC* segregation is asymmetric. This mode of chromosome segregation is somewhat similar to classical chromosome segregation described in other bacteria such as *C*. *crescentus* in which ParA segregates one of the daughter *oriC*s unidirectionally. However, even though in *Streptomyces* ParA is required for separation of the duplicated tip-proximal *oriC*s, the *oriC* does not move toward the tip and the distance between apical ParB/o*riC* complex and the tip remains constant during segregation. Unlike in *C*. *crescentus* and *V*. *cholerae*, ParA assemblies in *Streptomyces* were not observed to retract to the cell pole (tip). Those observations, and the increase of the distance between apical ParB/o*riC* complex and the tip during ParA overexpression, suggests a lower degree of ParA assembly dynamics in *Streptomyces* compared to rod-shaped bacteria. Thus, in *Streptomyces* ParA dependent *oriCs* separation is not a result of the active movement of one of them but rather is directly dependent on the tip extension, which suggests the anchorage model.

It was shown earlier that in *Streptomyces*, ParA interacts with the tip-associated, coiled-coil Scy protein, which together with DivIVA and FilP forms a polarisome complex (TIPOC) [18,29,32]. Until now, it was believed that the function of the polar complex was to maintain the rigidity of the extending tip and to establish the cell wall synthesis machinery [14,33]. We have revealed an additional function, which is to provide anchorage for the *oriC* of the apical chromosome. Presumably densely-packed protein complexes occupy the tip-proximal space occluding the chromosome from migrating to the very tip. The interaction between the polarisome, ParA and ParB complex assembled at *oriC* ensures that this chromosome closely follows the extending hyphae but also orientates the apical chromosome with its *oriC* toward the tip of the hyphae. Elimination of ParB moved the *oriC* region, but not the nucleoid, away from the tip, whereas the absence of ParA resulted in shifting of both the nucleoid and ParB complex away from the tip. This may be explained by potential interactions of tip-localized ParA with DNA (in this case, the tip proximal chromosome only), such as detected in other bacteria [34]. The apical anchorage of the segregation machinery is not unique to *Streptomyces*. In *C*. *crescentus*, polar proteins TipN and PopZ, mediate chromosome segregation via interactions with ParA and ParB [9,35–37]. In *V*. *cholerae oriC*I targeting to the cell poles is executed by the interaction between the polar protein HubP and ParA [10]. During *B*. *subtilis* sporulation, the origin-binding protein RacA is recruited by apically localized DivIVA, and during vegetative growth DivIVA may be involved in an indirect interaction (via MinD) with segregation proteins and *oriC* positioning [38,39]. Among the Actinobacteria, DivIVA interacts with ParA in *M*. *smegmatis*; however, the *oriC* is positioned close to center of cell, at least in optimal growth conditions [40], and the biological role of this interaction has not yet been described. In contrast to *M*. *smegmatis*, in *C*. *glutamicum oriC* is also anchored at the cell pole by the interaction between the ParB and the pole associated DivIVA [41,42]. Interestingly, in *S*. *coelicolor* we could never detect the direct interaction between ParB or ParA and DivIVA. In fact, the distance between ParB and the tip-localized DivIVA is likely to exclude this possibility. The subapical localization of *oriC* in *S*. *coelicolor*, is reminiscent of the *oriC* localization in *Myxococcus xanthus*, however the positioning of the *oriC* is presumably dependent on different mechanisms.

At this point it cannot be fully determined if the interaction of ParA with Scy in *Streptomyces* may be playing a similar role to the sequestration of monomeric ParA by TipN or PopZ in *C*. *crescentus*. Scy does not bind the ParAK44E mutant (ATP binding and dimerization abolished), and it promotes dissociation of higher order ParA assembly [29]. Microscopy analysis showed that ParA, at least transiently, co-localizes with Scy in the DNA-free region at the hyphal tip. A distinctive features of *Streptomyces* ParA is its ability to form higher order structures in the absence of DNA [29]. On the basis of these observations we speculate that ParA may extend from the Scy complex at the tip forming a higher order structure that interacts with ParB and anchors *oriC*. However, due to its higher stability, upon contact with the segrosome, ParA assemblies, do not retract and instead serve as the anchor for the apical ParB complex. The lack of direct interaction between ParB and Scy or DivIVA excludes the immobilization of the ParB complex independently of ParA. Moreover, we have shown here that in the strains with *parA* mutations, including a mutation that abolishes interaction with Scy and ParB, or in absence of Scy, the connection between the tip and the first chromosome was lost. Thus, the unique features of *Streptomyces* ParA, which presumably have evolved as an adaptation to hyphal growth, contribute to a less dynamic mechanism of chromosome segregation, involving the anchorage of a single *oriC* region exclusively.

During new branch formation, ParA recruited to the polarisome captures one of the multiple chromosomal *oriCs* complexed with ParB and directs it to the new hyphal tip. It was shown earlier that, DivIVA is required to establish a new hyphal branch and Scy organizes the new polarity center [17,18,43]. We have demonstrated that in strains lacking ParA and ParB, the branch or germ tube extension is frequently abolished. It was shown earlier that abortive branches are not populated by replisomes [27]. Here, we demonstrated that, in the *parA* and *parB* mutant strains, the length of the branch when it is populated with *oriC* is significantly increased and greater than the mean distance between *oriC* and the tip. This suggests that *parA* or *parB* deletion does not simply shift the *oriC* away from the tip but rather breaks its anchorage. We suggest that impaired new branch extension is the result of a delayed population of the new branch with the nucleoid(s). The eventual appearance of chromosomes in the empty branches is likely to be the result of diffusion from the crowded stems. The fact that *oriC* targeting to the branch does not need to directly follow *oriC* duplication proves that ParA mediated anchorage is not dependent on post-replicational segregation. It is tempting to speculate that the role of ParB complexes at the chromosomes along the hyphae is to facilitate targeting of chromosomes to the newly forming branches by interacting with ParA. This would represent a new function of ParA and polarisome complexes during germination and formation of new branches.

The establishment of new hyphal tips is yet another feature of *Streptomyces* growth that resembles filamentous fungi. Hyphal growth in fungi is driven by Spitzenkörper, the polar structure which assembles secretory vesicles delivered to the apical region by cytoskeletal tracks [44]. In *Aspergillus nidulans*, it was shown that although branch initiation is independent of the presence of a nucleus, the population of the extending branch with the nuclei is very efficient and dependent on the coiled-coil proteins ApsB and ApsA, which are also responsible for microtubule organization and nuclei migration [45–47]. Interestingly, in *Arabidopsis*, an increase in the distance between the nucleus and the root hair apex stops cell growth [48]. Considering that all apically growing organisms, including plant root hairs, pollen tubes, fungal hyphae and filamentous bacteria, require the assembly of cell wall building blocks at one cell pole (Spitzenkörper in fungi) [49,50], we conclude that there is a need for a machinery that assures the delivery of genetic material into the elongating cell and suggest that similar mechanisms permitting polar growth have evolved in both eukaryotic and bacterial cells.

The distribution of subapical chromosomes is rather random and we speculate that in the absence of cell division there is no requirement for their active segregation. In fact, the newly duplicated *oriC* in the hyphal stem are not separated after replication as efficiently as apical *oriCs*, as expected in the absence of ParA. Interestingly, we observed a flow of chromosomes following the extending tip, which indicated that although only the apical *oriC* is tightly anchored, the other chromosomes, also follow the extending hyphae. The flow of the replisomes in hyphae has been observed in *Streptomyces* before [27]; however, the mechanism of the movement of chromosomes remains unknown. It is possible that molecular crowding and viscoelastic properties of the environment of nucleoids and/or internucleoid linkages provide the cytoplasmic flow that pulls the chromosomes behind the tip in the apically extending cell. The nucleoid flow in extending hyphae is reminiscent of nuclear migration associated with hyphal growth in filamentous fungi. In polarized cells of filamentous fungi, the nuclei distribution is dependent on motor proteins but movement of the nuclei is regarded to be also partially passive and driven by cytoplasmic flow [45,51,52]. In addition, in apically extending plant root hairs, nuclei follow the tip at a constant distance to the cortex [48]. It is likely that hyphal growth may impose a similar pattern of chromosome migration in *Streptomyces*.

**Fig 7:**
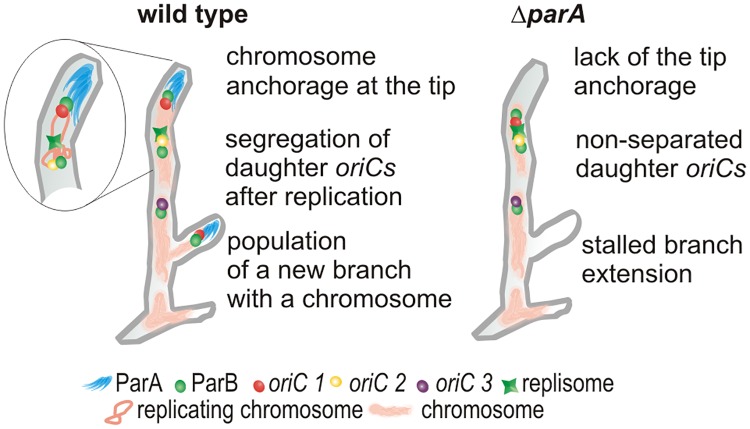
Model of ParA anchorage of the *oriC*/ParB complex at the tips of extending hyphae. Following chromosome replication, the tip-proximal of one of the two daughter *oriC*s is captured by the polarisome associated ParA which maintains its constant distance to the tip. The other daughter *oriC* remains associated with the replisome and is left behind by the extending tip. During branching the *oriC*/ParB complex proximal to newly established hyphal tip is captured by ParA and targeted into the branch.

In conclusion, our observations support a chromosomal anchor model (Fig 7) in which ParA interacts with a polarisome and binds one of the multiple *oriC*s associated with ParB. Soon after the initiation of apical chromosome replication, ParA captures one, tip-proximal, daughter *oriC* and maintains its constant distance to the tip. Remarkably the other daughter *oriC* is abandoned by the extending tip and allows that chromosome to act as the template for tip distal replication and chromosome population of branches. ParA also imposes the apical *oriC* orientation of the first chromosome. During the new hyphal tip establishment, ParA serves as a tip-anchor that captures one of the ParB-*oriC* complexes from multigenomic cellular compartments. Targeting the chromosome to the new hyphal tube permits its efficient extension. Thus, although the interaction of ParA with polar proteins as part of the chromosome segregation mechanism is shared by a number of bacterial species, in *Streptomyces*, this interaction provides a unique tip-anchor essential during spore germination and branching.

## Materials and Methods

### Bacterial strains growth conditions

The *E*. *coli* and *S*. *coelicolor* strains used are listed in S1 Table. DNA manipulations were carried out by standard protocols [53]. Culture conditions, antibiotic concentrations, transformation and conjugation methods followed general procedures for *E*. *coli* [53] and *Streptomyces* [54]. The *oriC* region of *S*. *coelicolor* M145 was labelled with Tn*5341* carrying 120 tandemly arrayed copies of *tetO* and the apramycin resistance gene. Following *in vitro* transposition of cosmid SCH18 with Tn*5431* [31,55], an obtained cosmid (EJTH31A) carrying the transposon at a site located in an intergenic region ~29 kb from the *oriC* region was selected. Introduction of the EJTH31A cosmid into *S*. *coelicolor* M145 generated the strain, EJTH31. This strain was further modified by introduction of the integrating plasmid pMS83-mCherry to express *tetRmcherry* fusion yielding the “wild type” FROS strain, DJ-NL102 (S1A Fig) (for more details, see Supplementary Information). To avoid potential selection for chromosomal rearrangements or deletions of the *tetO* cassette, the FROS strain was always cultured in the presence of 0.1 μg ml^-1^ anhydrotetracycline, except for microscopy analyses of the *oriC* localization. A detailed description of other strains construction is presented in the Supplementary Information.

### Microscopy analysis

For the fixed microscopy specimen preparation, spores were inoculated in the acute-angled junction of coverslips inserted at 45° in MM agar containing 1% mannitol [54] and cultured for 21–24 h. For ParA induction, the strain DJ532 was grown in the presence of thiostrepton (5 μg ml^-1^). Staining procedures were as described previously [21,56]. Briefly, samples were fixed for 10 min with paraformaldehyde/glutaraldehyde mixture, digested 2 min with 1 mg ml^-1^ lysozyme, washed with PBS and blocked with 2% BSA. For immunostaining, samples were incubated with antibody against ParA (1:5000 dilution) overnight, washed six times with PBS and then incubated for 1 h with secondary antibody (anti-rabbit) conjugated with Alexa Fluor546. For DNA staining, samples were incubated with 1–10 μg ml^-1^ propidium iodide, and for cell wall visualization with 1–10 μg ml^-1^ WGA-Texas red or Alexa Fluor350 conjugate (Molecular Probes). After five washes with PBS, coverslips were mounted in 50% glycerol in PBS buffer. Florescence microscopy was carried out using a Zeiss AxioImager M1 or Zeiss Observer Z1 with camera AxioCam MRm Rev. The images were analyzed by AxioVision Rel. 4.5 Software equipped with AutoMeasure module or FIJI Software. R analysis tool was used for foci detection [57]. The focus was identified when the fluorescence intensity was above the threshold, which was set as 50% of the highest signal intensity in the particular hyphae.

For time-lapse imaging, spore dilutions were spotted onto cellophane membranes on MM solid medium supplemented with 1% mannitol and cultured for 2 h (for germination analysis) or 24 h (for vegetative growth analysis) before the start of the experiment. The cellophane membrane was transferred to a μ-dish (∅35 mm, Ibidi) and covered with a block of agar. Fluorescence microscopy was carried out at 30°C, using a Delta Vision Elite inverted microscope equipped with a 100x oil-immersion objective, ultimate focus and Olympus IX71 camera. Images were acquired every 10 minutes using DIC (differential interference contrast) and EGFP or cherry filter set with the exposition time of 50 and 100 or 200 ms, respectively. Images were analyzed using FIJI software. Data analysis was performed using R software. The cell contour was determined manually in DIC images. After background signal subtraction, the fluorescence along the hyphae was averaged using Fiji software and subsequently R package Peaks was used to find and localize foci [58]. For every time-point, a fluorescence intensity profile for the whole hyphae was generated. Based on the raw data a smoothed intensity profile was calculated using R package Peaks with a Markov chain method. All maxima indicated by the algorithm were manually checked and false positives were discarded. This approach allowed us to determine the exact position of all foci in the hyphae. If two maxima were observed that could be distinguished by the Peaks package, then we assumed that two foci were present in the hyphae (S3A, S3B and S3C Fig). Density (used for distribution analysis) was calculated using a kernel density estimate in R program [57]. ANOVA, Games-Howell test (Figs 3, 4 and 6), and chi-squared test (Fig 6) were applied for statistical analysis. Differences were considered significant when p-values were lower than 0.05.

## Supporting Information

S1 Fig Construction and verification of the FROS strains. Scheme of construction of the FROS strain (DJ-NL102).Southern blotting verification of the FROS strains. The chromosomal DNA was digested with EcoRI and the probes were designed as shown in the scheme on the right.Verification of TetR-mCherry, DnaN-EGFP and ParB-EGFP fluorescence in analyzed strains. The cell extracts were analyzed in semi-denatured SDS-PAGE (without heating the samples) and fluorescence was detected using molecular imager PharosFX system.
(PDF)

S2 Fig Growth and chromosome replication are not disturbed in the FROS strain. FROS foci in DJ-NL102 strain hyphae and their disappearance in the presence of 0.1 μg ml^-1^anhydrotetracycline (aTC), scale bar—5 μm.The distances between FROS foci in FROS strain (DJ-NL102) hyphae (20 hyphae measured).Growth rate of wild type (M145), FROS strain (DJ-NL102) and FROS *dnaN-egfp* (AK122) strain. Experiments were performed using a Bioscreen-C (Automated Growth Curves Analysis System, Growth Curves USA) with five replicates for each strain. OD_600_ of cultures was measured every 10 minutes for 60 hours at 30°C and the averaged results of replicates were plotted.Number of replisomes in the hyphae of FROS *dnaN-egfp* (AK122) and control strain *dnaN-egfp* (J3337) in relation to hyphae length.Distribution of replisomes in the hyphae of FROS *dnaN-egfp* (AK122) and control strain *dnaN-egfp* (J3337).Images showing the replisomes in hyphae of FROS *dnaN-egfp* (AK122) and control strain *dnaN-egfp* (J3337), scale bar—5 μm.
Panels B, D, E show conventional boxplots with median and the lower and upper "hinges" that correspond to the first and third quartiles, all observations are marked as semitransparent points.
(PDF)

S3 Fig The analysis of FROS foci positions in time-lapse microscopy images of FROS strain (DJ-NL102) germinating spores and extending vegetative hyphae. Time-lapse snapshots taken every 10 minutes of the germinating spores of FROS strain (DJ-NL102). The images are the overlay of TetR-mCherry fluorescence and DIC image (gray), scale bar - 1 μm. The Fiji program was used to subtract background from red channel and hyphal boundaries were manually defined.Line plot showing fluorescence intensity profile and smoothed intensity profile (black and blue lines) generated for each image. Smoothed intensity profile was calculated on the basis of the raw data, using R package Peaks with a Markov chain method. All maxima indicated by the algorithm were manually checked and false positives were discarded.Representation of hyphae (grey bar) with identified fluorescence maxima (red points).Time-lapse snapshots of FROS strain (DJ-NL102) germinating spore (top panel) and vegetative hypha (bottom panel). The images show separate channels: TetR-mCherry fluorescence (red) in the hyphal outline and DIC images (gray), as indicated. Scale bar—1 μm.
(PDF)

S4 Fig The localization of ParA (green) overlaid with DNA (red) in hyphae of *parA-egfp* strain (DJ590). In vegetative hyphae (marked with “v”), ParA localized exclusively at the hyphal tips while in sporogenic hyphae (marked with „s”) it was dispersed along the hyphae. The images are the snapshots of fixed 24 hours hyphae stained with propidium iodide (PI) to visualize DNA. Scale bar– 5 μm.
(PDF)

S5 Fig Distance of the stained nucleoid from the tip in wild type M145, Δ*parA* (J3306) and Δ*parB* (J3305) strains. Red crossbars show mean (measured for 370–418 hyphae) with 95% confidence intervals.
(PDF)

S6 Fig Time-lapse analysis of the FROS complexes in extending hyphae of “wild type” FROS (DJ-NL102), Δ*parA* FROS (AK115) and Δ*parB* FROS (AK114) strains. Position of the FROS complexes in the extending hyphae. Grey bars are representations of the extending hyphae with 95% confidence intervals for hyphal length and semitransparent colored dots represent *oriC* positions (red–*oriC* 1, yellow–*oriC* 2, purple–*oriC* 3) analyzed in 41 hyphae of DJ-NL102, 31 of AK115 and 30 of AK114 strain, colored lines indicate 95% mean confidence intervals.Distribution (shown as probability density function) of the distances between the hyphal tip and *oriC* 1 (red), *oriC* 2 (yellow) and *oriC* 3 (purple) along the hyphae of “wild type” FROS (DJ-NL102), **Δ***parA* FROS (AK115) and **Δ***parB* FROS (AK114) strains.
(PDF)

S7 Fig Distance of ParB-EGFP from the hyphal tip in "wild type” (J3310) and Δ*scy* (BD05) during growth analyzed from the time of branch emergence (time 0). The line shows the mean with 95% confidence intervals indicated by the green area. 40 hyphae of J3310 strain, and 31 hyphae of BD05 were measured.
(PDF)

S8 Fig Time-lapse snapshots of FROS (TetR-mCherry fluorescence, red) and DnaN-EGFP foci (green) in the extending hyphae of “wild type” FROS *dnaN-egfp* (AK122) (top panel) and Δ*parA* FROS *dnaN-egfp* (AK123) (bottom panel) strains. The images show separate channels: TetR-mCherry fluorescence (red) and DnaN-EGFP (green) in the hyphal outline and DIC images (grey), scale bar—1 μm.
(PDF)

S9 Fig ParB binds daughter *oriCs* soon after duplication. Time-lapse snapshots of AK101 hyphae, showing in separate channels DIC image, ParB-EGPFP (green) and DnaN-mCherry (red) foci, scale bar—1 μm.Time of detection of duplicated ParB complexes (blue bars) and FROS signal (red bars) after replisome appearance. Error bars show 95% confidence intervals. The analysis was performed for 29 hyphae of FROS *dnaN-egfp* strain (AK122) and 33 hyphae of *parB-egfp dnaN-mcherry* (AK101) strain.
(PDF)

S10 Fig Distance between tip-proximal and stem (tip-distal) FROS foci at the indicated time after their duplication in “wild type” FROS dnaN-egfp (AK122). Crossbars show the mean with 95% confidence intervals. The analysis was performed 27 stem and 32 tip-proximal FROS complexes.
(PDF)

S11 Fig Time-lapse snapshots of FROS (TetR-mCherry fluorescence, red) in the germinating spores (top panel) and branching hyphae (bottom panel) of “wild type” FROS (DJ-NL102) and Δ*parA* FROS (AK115) strains. The images show separate channels: TetR-mCherry fluorescence (red) in the hyphae outlined and DIC images (grey), scale bar—1 μm.
(PDF)

S1 Movie The tip-proximal chromosome follows the extending tip. Time-lapse analysis of FROS strain (DJ-NL102).
(MOV)

S2 Movie *oriC* is captured at the tip soon after replication. Time-lapse analysis of FROS *dnaN-egfp* strain (AK122).
(MOV)

S3 Movie In Δ*parA* strain *oriC* fails to be captured after duplication. Time-lapse analysis of **Δ***parA* FROS *dnaN-egfp* strain (AK123).
(MOV)

S4 Movie The localization of *oriC* shows the population of the emerging branch with the chromosome. Time-lapse analysis of FROS strain (DJ-NL102).
(MOV)

S5 Movie The population of the emerging branch with chromosome is disturbed and branch extension is abolished in Δ*parA* strain. Time-lapse analysis of **Δ***parA* FROS strain (AK115).
(MOV)

S1 Table Strains and plasmids used in this study. (PDF)

S2 Table Oligonucleotides used in this study. (PDF)

S1 Text Supplementary Materials and Methods. (PDF)
