# Supplementary material for: Unique Function of the Bacterial Chromosome Segregation Machinery in Apically Growing Streptomyces - Targeting the Chromosome to New Hyphal Tubes and its Anchorage at the Tips
Source: PLoS Genet. 2016 Dec 15;12(12):e1006488. doi: 10.1371/journal.pgen.1006488 (PMC5157956; doi:10.1371/journal.pgen.1006488)
Supplement: S1 Fig — Scheme of construction of the FROS strain (DJ-NL102).Southern blotting verification of the FROS strains. The chromosomal DNA was digested with EcoRI and the probes were designed as shown in the scheme on the right.Verification of TetR-mCherry, DnaN-EGFP and ParB-EGFP fluorescence in analyzed strains. The cell extracts were analyzed in semi-denatured SDS-PAGE (without heating the samples) and fluorescence was detected using molecular imager PharosFX system. (PDF) [file pgen.1006488.s001.pdf]

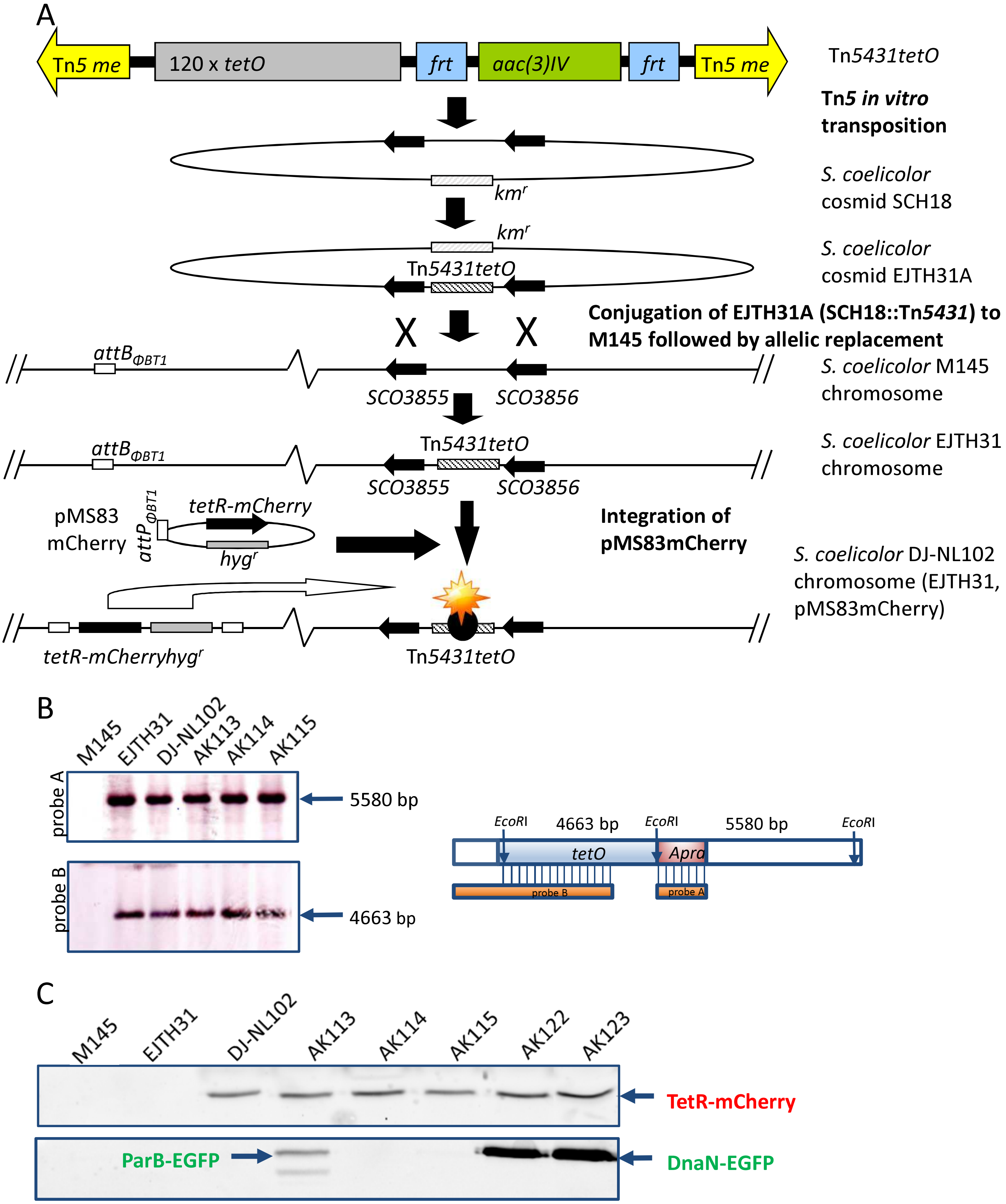

Fig. S1 Construction and verification of the FROS strains.

A. Scheme of construction of the FROS strain (DJ-NL102).
B. Southern blotting verification of the FROS strains. The chromosomal DNA was digested with EcoRI and the probes were designed as shown in the scheme on the right.
C. Verification of TetR-mCherry, DnaN-EGFP and ParB-EGFP fluorescence in analyzed strains. The cell extracts were analyzed in semi-denatured SDS-PAGE (without heating the samples) and fluorescence was detected using molecular imager PharosFX system.
