# Supplementary material for: Unique Function of the Bacterial Chromosome Segregation Machinery in Apically Growing Streptomyces - Targeting the Chromosome to New Hyphal Tubes and its Anchorage at the Tips
Source: PLoS Genet. 2016 Dec 15;12(12):e1006488. doi: 10.1371/journal.pgen.1006488 (PMC5157956; doi:10.1371/journal.pgen.1006488)
Supplement: S2 Fig — FROS foci in DJ-NL102 strain hyphae and their disappearance in the presence of 0.1 μg ml-1anhydrotetracycline (aTC), scale bar—5 μm.The distances between FROS foci in FROS strain (DJ-NL102) hyphae (20 hyphae measured).Growth rate of wild type (M145), FROS strain (DJ-NL102) and FROS dnaN-egfp (AK122) strain. Experiments were performed using a Bioscreen-C (Automated Growth Curves Analysis System, Growth Curves USA) with five replicates for each strain. OD600 of cultures was measured every 10 minutes for 60 hours at 30°C and the averaged results of replicates were plotted.Number of replisomes in the hyphae of FROS dnaN-egfp (AK122) and control strain dnaN-egfp (J3337) in relation to hyphae length.Distribution of replisomes in the hyphae of FROS dnaN-egfp (AK122) and control strain dnaN-egfp (J3337).Images showing the replisomes in hyphae of FROS dnaN-egfp (AK122) and control strain dnaN-egfp (J3337), scale bar—5 μm. Panels B, D, E show conventional boxplots with median and the lower and upper "hinges" that correspond to the first and third quartiles, all observations are marked as semitransparent points. (PDF) [file pgen.1006488.s002.pdf]

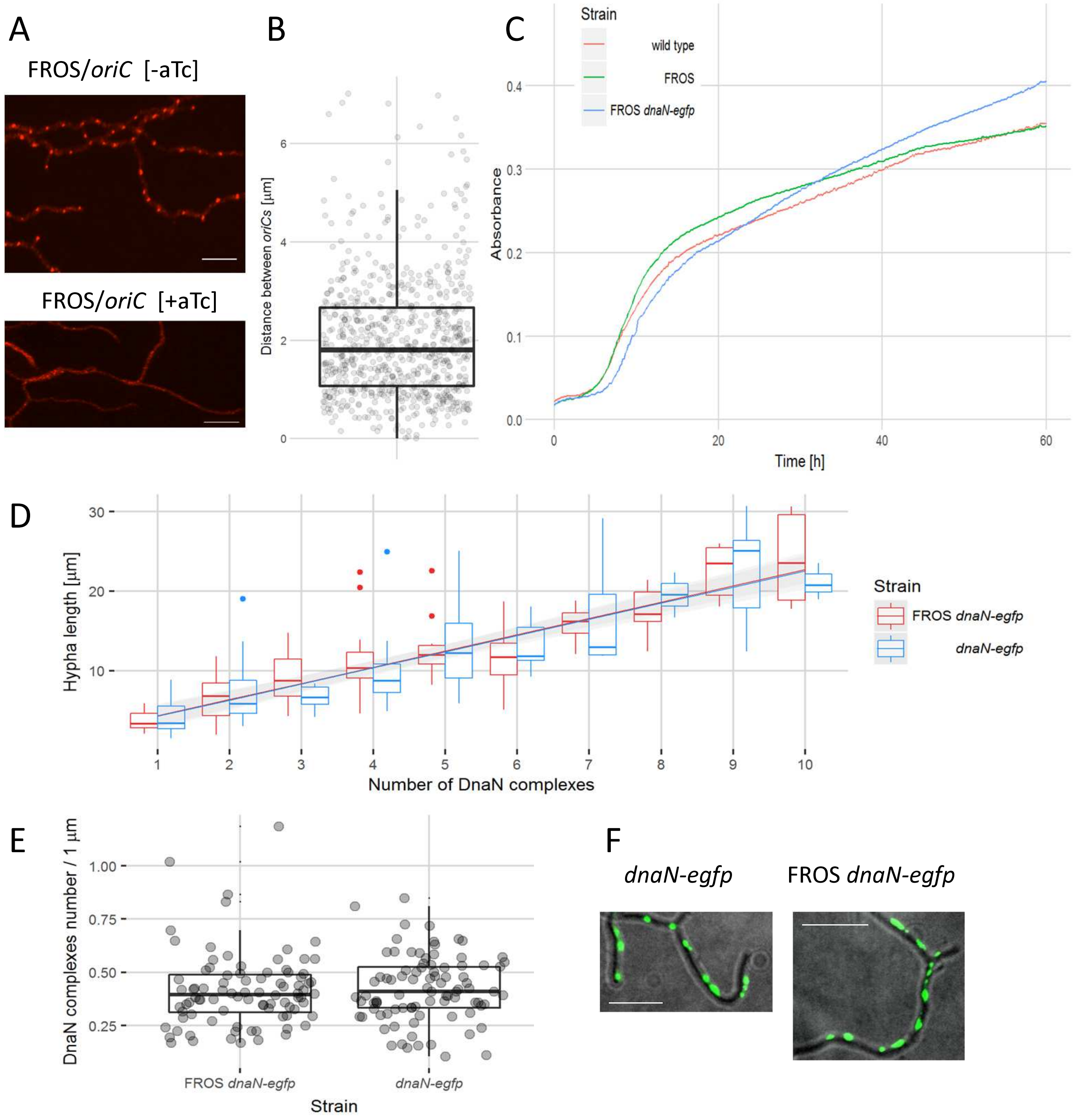

Fig. S2 Growth and chromosome replication are not disturbed in the FROS strain.

A. FROS foci in DJ-NL102 strain hyphae and their disappearance in the presence of 0.1 mg ml^{-1} anhydrotetracycline (aTC), scale bar - 5 µm.

B. The distances between FROS foci in FROS strain (DJ-NL102) hyphae (20 hyphae measured).

C. Growth rate of wild type (M145), FROS strain (DJ-NL102) and FROS *dnaN-egfp* (AK122) strain. Experiments were performed using a Bioscreen-C (Automated Growth Curves Analysis System, Growth Curves USA) with five replicates for each strain. OD_{600} of cultures was measured every 10 minutes for 60 hours at 30°C and the averaged results of replicates were plotted.

D. Number of replisomes in the hyphae of FROS *dnaN-egfp* (AK122) and control strain *dnaN-egfp* (J3337) in relation to hyphae length.

E. Distribution of replisomes in the hyphae of FROS *dnaN-egfp* (AK122) and control strain *dnaN-egfp* (J3337).

F. Images showing the replisomes in hyphae of FROS *dnaN-egfp* (AK122) and control strain *dnaN-egfp* (J3337), scale bar - 5 µm.

Panels B, D, E show conventional boxplots with median and the lower and upper "hinges" that correspond to the first and third quartiles, all observations are marked as semitransparent points.
