# Supplementary material for: Unique Function of the Bacterial Chromosome Segregation Machinery in Apically Growing Streptomyces - Targeting the Chromosome to New Hyphal Tubes and its Anchorage at the Tips
Source: PLoS Genet. 2016 Dec 15;12(12):e1006488. doi: 10.1371/journal.pgen.1006488 (PMC5157956; doi:10.1371/journal.pgen.1006488)
Supplement: S3 Fig — Time-lapse snapshots taken every 10 minutes of the germinating spores of FROS strain (DJ-NL102). The images are the overlay of TetR-mCherry fluorescence and DIC image (gray), scale bar - 1 μm. The Fiji program was used to subtract background from red channel and hyphal boundaries were manually defined.Line plot showing fluorescence intensity profile and smoothed intensity profile (black and blue lines) generated for each image. Smoothed intensity profile was calculated on the basis of the raw data, using R package Peaks with a Markov chain method. All maxima indicated by the algorithm were manually checked and false positives were discarded.Representation of hyphae (grey bar) with identified fluorescence maxima (red points).Time-lapse snapshots of FROS strain (DJ-NL102) germinating spore (top panel) and vegetative hypha (bottom panel). The images show separate channels: TetR-mCherry fluorescence (red) in the hyphal outline and DIC images (gray), as indicated. Scale bar—1 μm. (PDF) [file pgen.1006488.s003.pdf]

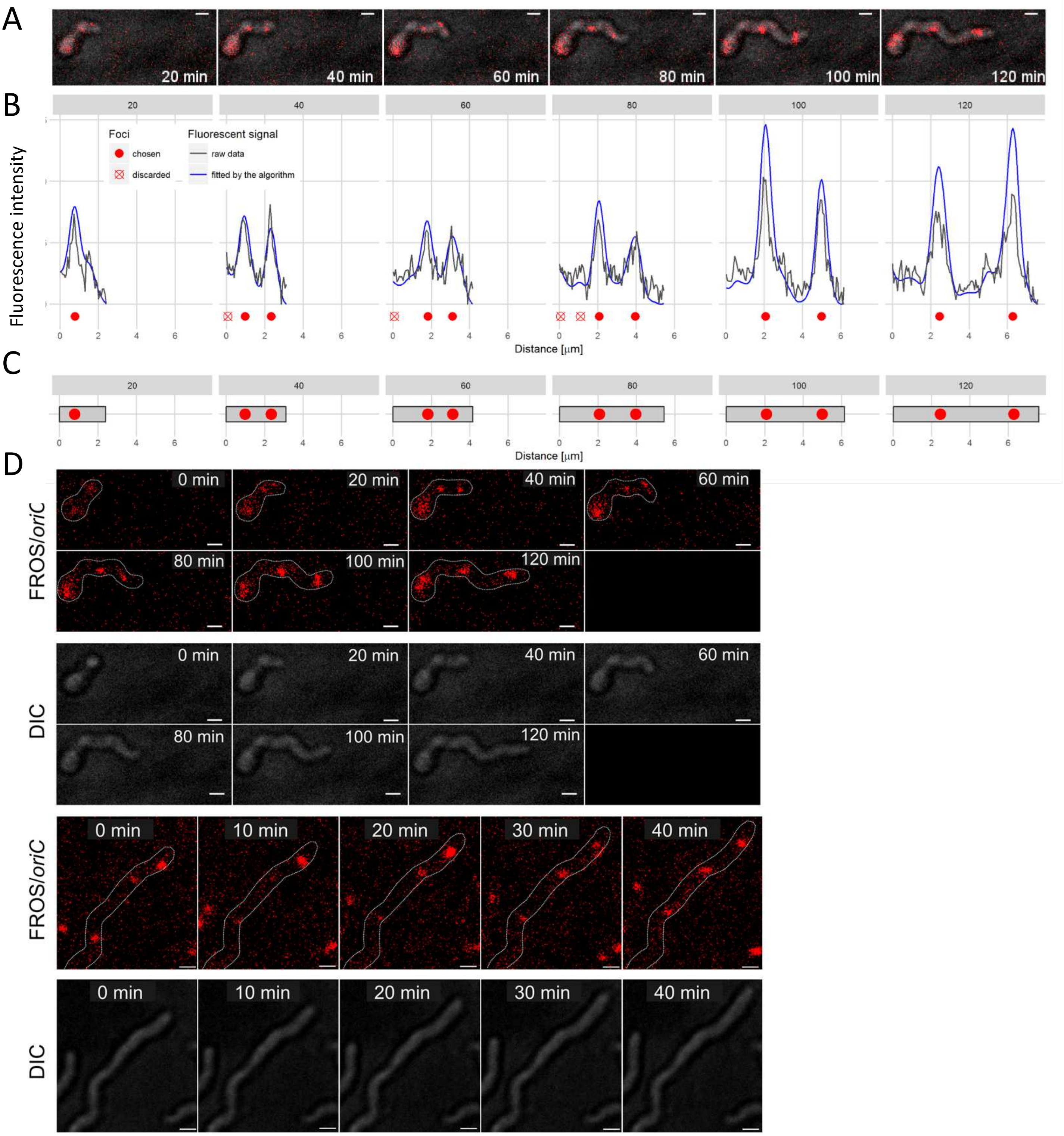

Fig. S3 The analysis of FROS foci positions in time-lapse microscopy images of FROS strain (DJ-NL102) germinating spores and extending vegetative hyphae.

A. Time-lapse snapshots taken every 10 minutes of the germinating spores of FROS strain (DJ-NL102). The images are the overlay of TetR-mCherry fluorescence and DIC image (gray), scale bar -1 µm. Fiji program was used to subtract background from red channel and hyphae boundaries were manually defined.

B. Line plot showing fluorescence intensity profile and smoothed intensity profile (black and blue lines) generated for each image. Smoothed intensity profile was calculated on the basis of the raw data, using R package Peaks with a Markov chain method. All maxima indicated by the algorithm were manually checked and false positives were discarded.

C. Representation of hyphae (grey bar) with identified fluorescence maxima (red points).

D. Time-lapse snapshots of FROS strain (DJ-NL102) germinating spore (top panel) and vegetative hypha (bottom panel). The images show separate channels: TetR-mCherry fluorescence (red) in the hyphal outline and DIC images (gray), as indicated. Scale bar - 1 µm.
