# Supplementary material for: Unique Function of the Bacterial Chromosome Segregation Machinery in Apically Growing Streptomyces - Targeting the Chromosome to New Hyphal Tubes and its Anchorage at the Tips
Source: PLoS Genet. 2016 Dec 15;12(12):e1006488. doi: 10.1371/journal.pgen.1006488 (PMC5157956; doi:10.1371/journal.pgen.1006488)
Supplement: S4 Fig — In vegetative hyphae (marked with “v”), ParA localized exclusively at the hyphal tips while in sporogenic hyphae (marked with „s”) it was dispersed along the hyphae. The images are the snapshots of fixed 24 hours hyphae stained with propidium iodide (PI) to visualize DNA. Scale bar– 5 μm. (PDF) [file pgen.1006488.s004.pdf]

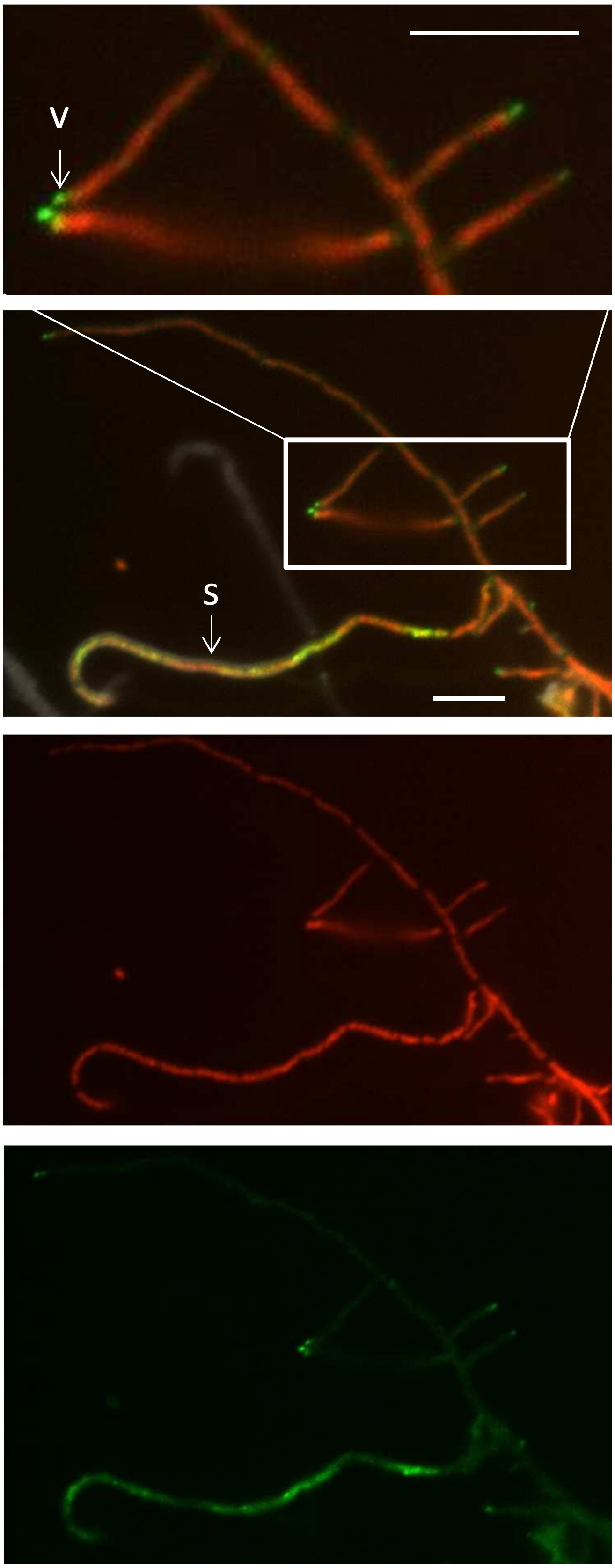

DNA (PI) /ParA-EGFP

DNA (PI) /ParA-EGFP

DNA (PI)

ParA-EGFP

Fig. S4 The localization of ParA (green) overlaid with DNA (red) in hyphae of *parA-egfp* strain (DJ590). In vegetative hyphae (marked with "v") ParA is localised exclusively at the hyphal tips while in sporogenic hyphae (marked with „s") it is dispersed along the hyphae. The images are the snapshots of fixed 24 hours hyphae stained with propidium iodide (PI) to visualise DNA. Scale bar – 5 μm.
