# Supplementary material for: Unique Function of the Bacterial Chromosome Segregation Machinery in Apically Growing Streptomyces - Targeting the Chromosome to New Hyphal Tubes and its Anchorage at the Tips
Source: PLoS Genet. 2016 Dec 15;12(12):e1006488. doi: 10.1371/journal.pgen.1006488 (PMC5157956; doi:10.1371/journal.pgen.1006488)
Supplement: S5 Fig — Red crossbars show mean (measured for 370–418 hyphae) with 95% confidence intervals. (PDF) [file pgen.1006488.s005.pdf]

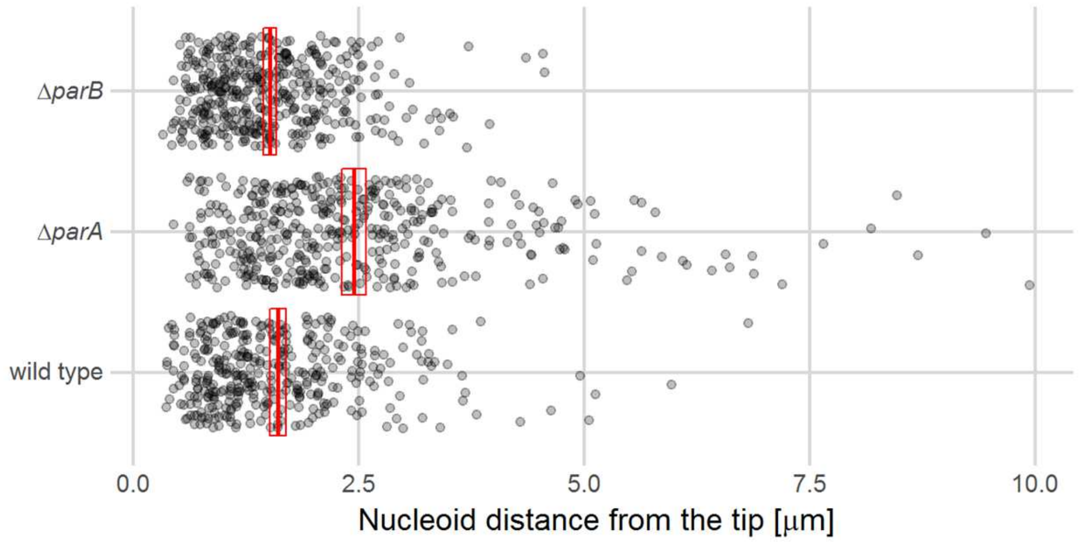

Fig. S5 Distance of the stained nucleoid from the tip in wild type M145, Δ*parA* (J3306) and Δ*parB* (J3305) strains. Red crossbars show mean (measured for 370-418 hyphae) with 95% confidence intervals.
