# Supplementary material for: Unique Function of the Bacterial Chromosome Segregation Machinery in Apically Growing Streptomyces - Targeting the Chromosome to New Hyphal Tubes and its Anchorage at the Tips
Source: PLoS Genet. 2016 Dec 15;12(12):e1006488. doi: 10.1371/journal.pgen.1006488 (PMC5157956; doi:10.1371/journal.pgen.1006488)
Supplement: S6 Fig — Position of the FROS complexes in the extending hyphae. Grey bars are representations of the extending hyphae with 95% confidence intervals for hyphal length and semitransparent colored dots represent oriC positions (red–oriC 1, yellow–oriC 2, purple–oriC 3) analyzed in 41 hyphae of DJ-NL102, 31 of AK115 and 30 of AK114 strain, colored lines indicate 95% mean confidence intervals.Distribution (shown as probability density function) of the distances between the hyphal tip and oriC 1 (red), oriC 2 (yellow) and oriC 3 (purple) along the hyphae of “wild type” FROS (DJ-NL102), ΔparA FROS (AK115) and ΔparB FROS (AK114) strains. (PDF) [file pgen.1006488.s006.pdf]

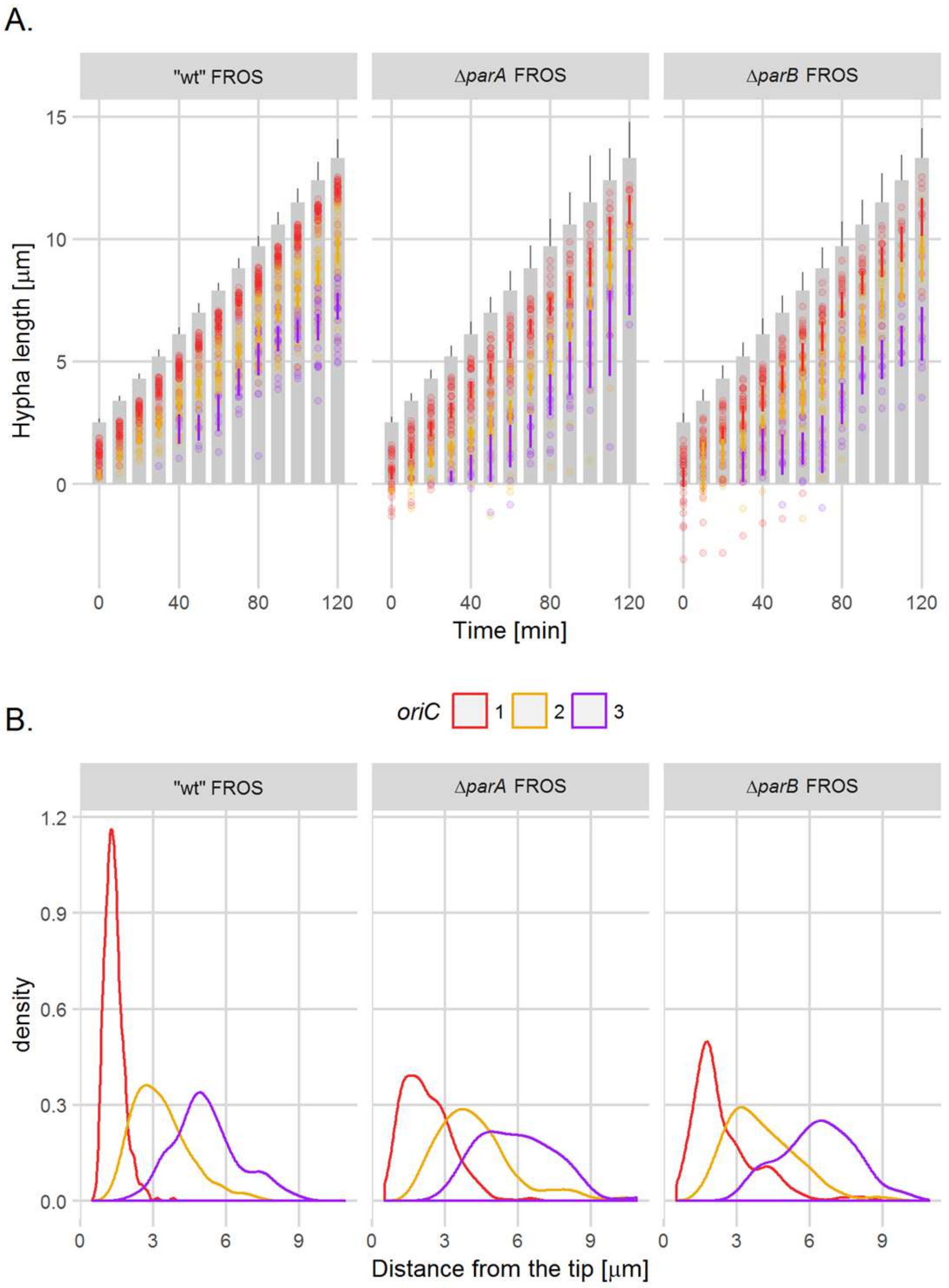

Fig. S6 Time-lapse analysis of the FROS complexes in extending hyphae of "wild type" FROS (DJ-NL102), Δ*parA* FROS (AK115) and Δ*parB* FROS (AK114) strains.

A. Position of the FROS complexes in the extending hyphae. Grey bars are representations of the extending hyphae with 95% confidence intervals for hyphal length and semitransparent colored dots represent *oriC* positions (red – *oriC* 1, yellow – *oriC* 2, purple – *oriC* 3) analyzed in 41 hyphae of DJ-NL102, 31 of AK115 and 30 of AK114 strain, colored lines indicate 95% mean confidence intervals.

B. Distribution (shown as probability density function) of the distances between the hyphal tip and *oriC* 1 (red), *oriC* 2 (yellow) and *oriC* 3 (purple) along the hyphae of "wild type" FROS (DJ-NL102), Δ*parA* FROS (AK115) and Δ*parB* FROS (AK114) strains.
