# Supplementary material for: Unique Function of the Bacterial Chromosome Segregation Machinery in Apically Growing Streptomyces - Targeting the Chromosome to New Hyphal Tubes and its Anchorage at the Tips
Source: PLoS Genet. 2016 Dec 15;12(12):e1006488. doi: 10.1371/journal.pgen.1006488 (PMC5157956; doi:10.1371/journal.pgen.1006488)
Supplement: S7 Fig — The line shows the mean with 95% confidence intervals indicated by the green area. 40 hyphae of J3310 strain, and 31 hyphae of BD05 were measured. (PDF) [file pgen.1006488.s007.pdf]

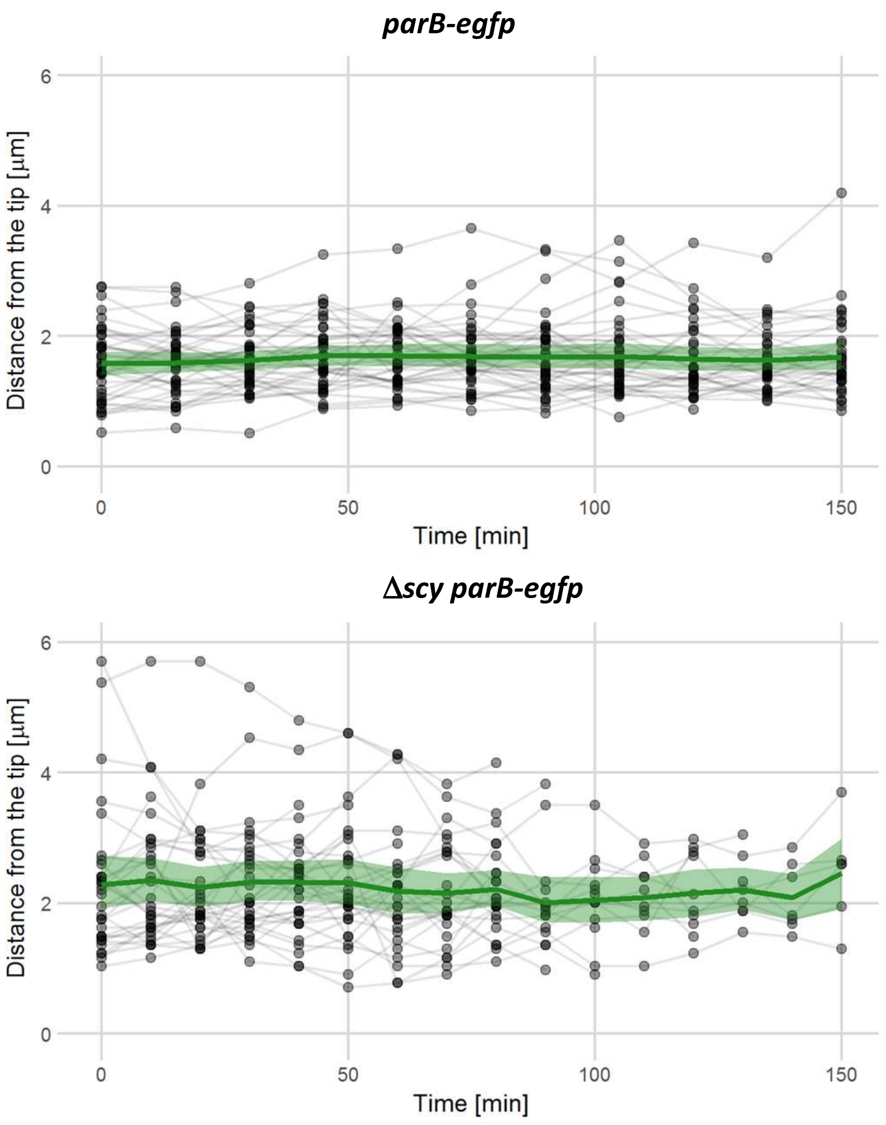

Fig. S7 Distance of ParB-EGFP from the hyphal tip in "wild type" (J3310) and Δ*scy* (BD05) during growth analyzed from the time of branch emergence (time 0). The line shows the mean with 95% confidence intervals indicated by the green area. 40 hyphae of J3310 strain, and 31 hyphae of BD05 were measured.
