# Supplementary material for: Unique Function of the Bacterial Chromosome Segregation Machinery in Apically Growing Streptomyces - Targeting the Chromosome to New Hyphal Tubes and its Anchorage at the Tips
Source: PLoS Genet. 2016 Dec 15;12(12):e1006488. doi: 10.1371/journal.pgen.1006488 (PMC5157956; doi:10.1371/journal.pgen.1006488)
Supplement: S8 Fig — The images show separate channels: TetR-mCherry fluorescence (red) and DnaN-EGFP (green) in the hyphal outline and DIC images (grey), scale bar—1 μm. (PDF) [file pgen.1006488.s008.pdf]

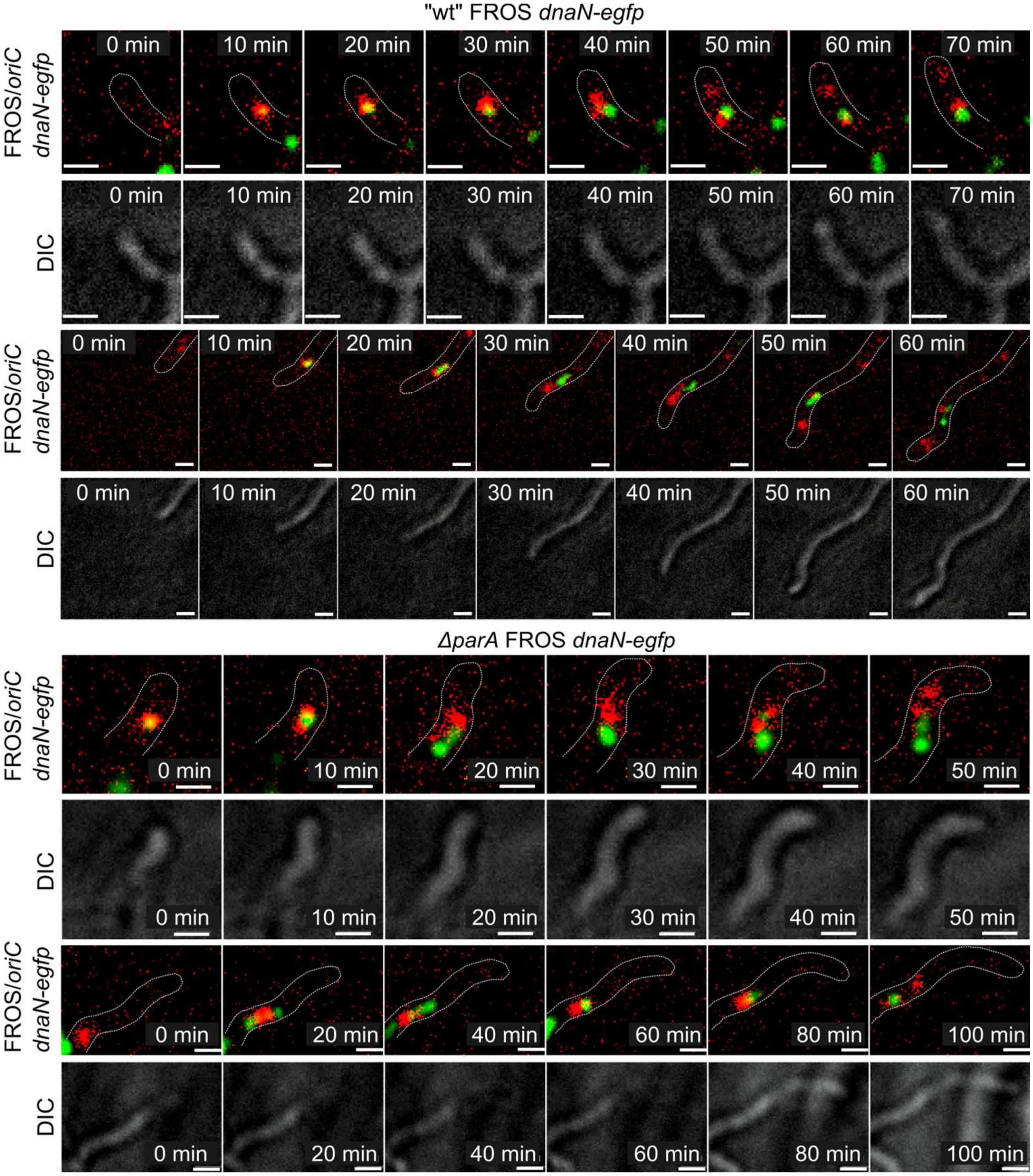

Fig. S8 Time-lapse snapshots of FROS (TetR-mCherry fluorescence, red) and DnaN-EGFP foci (green) in the extending hyphae of "wild type" FROS *dnaN-egfp* (AK122) (top panel) and Δ*parA* FROS *dnaN-egfp* (AK123) (bottom panel) strains. The images show separate channels: TetR-mCherry fluorescence (red) and DnaN-EGFP (green) in the hyphal outline and DIC images (grey), scale bar - 1 μm.
