# Supplementary material for: Unique Function of the Bacterial Chromosome Segregation Machinery in Apically Growing Streptomyces - Targeting the Chromosome to New Hyphal Tubes and its Anchorage at the Tips
Source: PLoS Genet. 2016 Dec 15;12(12):e1006488. doi: 10.1371/journal.pgen.1006488 (PMC5157956; doi:10.1371/journal.pgen.1006488)
Supplement: S9 Fig — Time-lapse snapshots of AK101 hyphae, showing in separate channels DIC image, ParB-EGPFP (green) and DnaN-mCherry (red) foci, scale bar—1 μm.Time of detection of duplicated ParB complexes (blue bars) and FROS signal (red bars) after replisome appearance. Error bars show 95% confidence intervals. The analysis was performed for 29 hyphae of FROS dnaN-egfp strain (AK122) and 33 hyphae of parB-egfp dnaN-mcherry (AK101) strain. (PDF) [file pgen.1006488.s009.pdf]

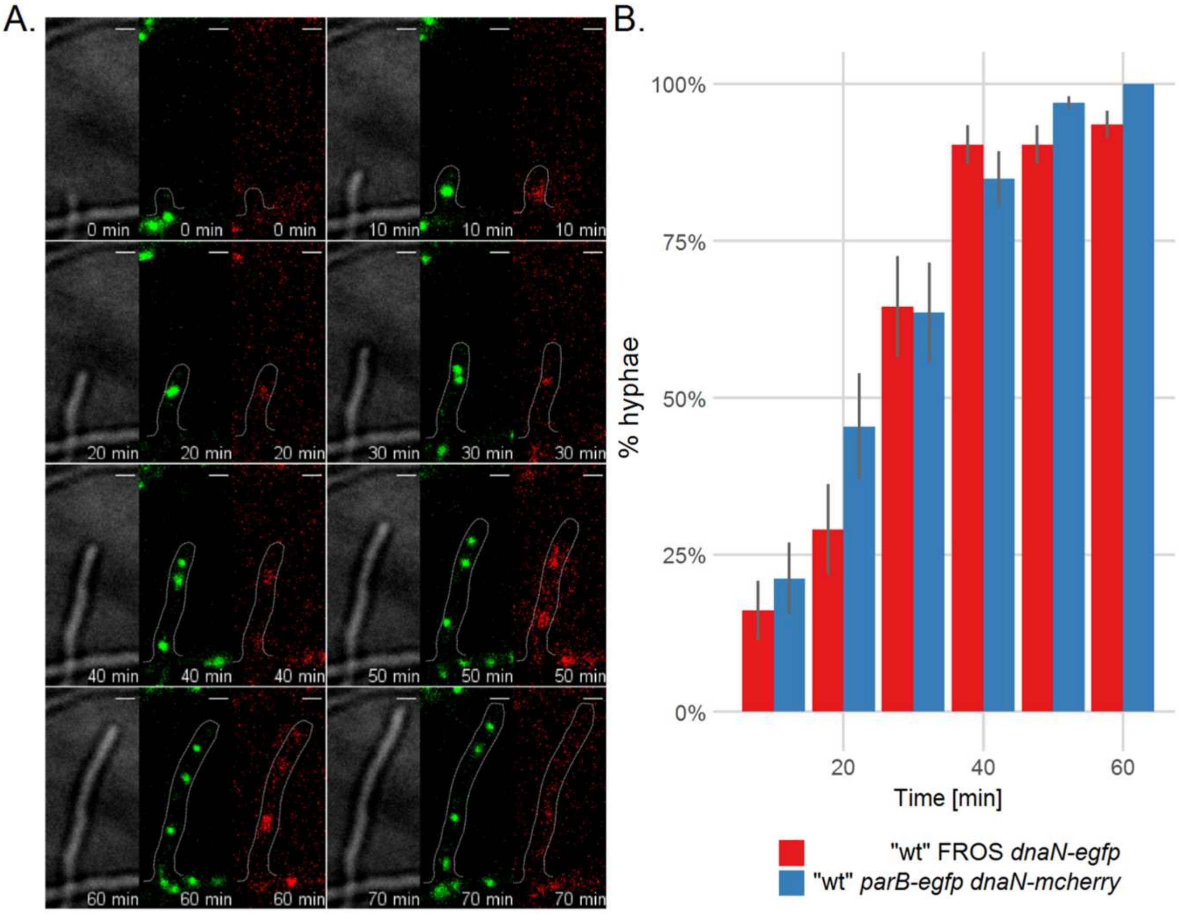

Fig. S9 ParB binds daughter *oriCs* soon after duplication

A. Time-lapse snapshots of AK101 hyphae, showing in separate channels: DIC image, ParB-EGPFP (green) and DnaN-mCherry (red), scale bar - 1 μm.

B. Time of detection of duplicated ParB complexes (blue bars) and FROS signal (red bars) after replisome appearance. Error bars show 95% confidence intervals. The analysis was performed for 29 hyphae of FROS *dnaN-egfp* strain (AK122) and 33 hyphae of *parB-egfp dnaN-mcherry* (AK101) strain.
