# Supplementary material for: Unique Function of the Bacterial Chromosome Segregation Machinery in Apically Growing Streptomyces - Targeting the Chromosome to New Hyphal Tubes and its Anchorage at the Tips
Source: PLoS Genet. 2016 Dec 15;12(12):e1006488. doi: 10.1371/journal.pgen.1006488 (PMC5157956; doi:10.1371/journal.pgen.1006488)
Supplement: S10 Fig — Crossbars show the mean with 95% confidence intervals. The analysis was performed 27 stem and 32 tip-proximal FROS complexes. (PDF) [file pgen.1006488.s010.pdf]

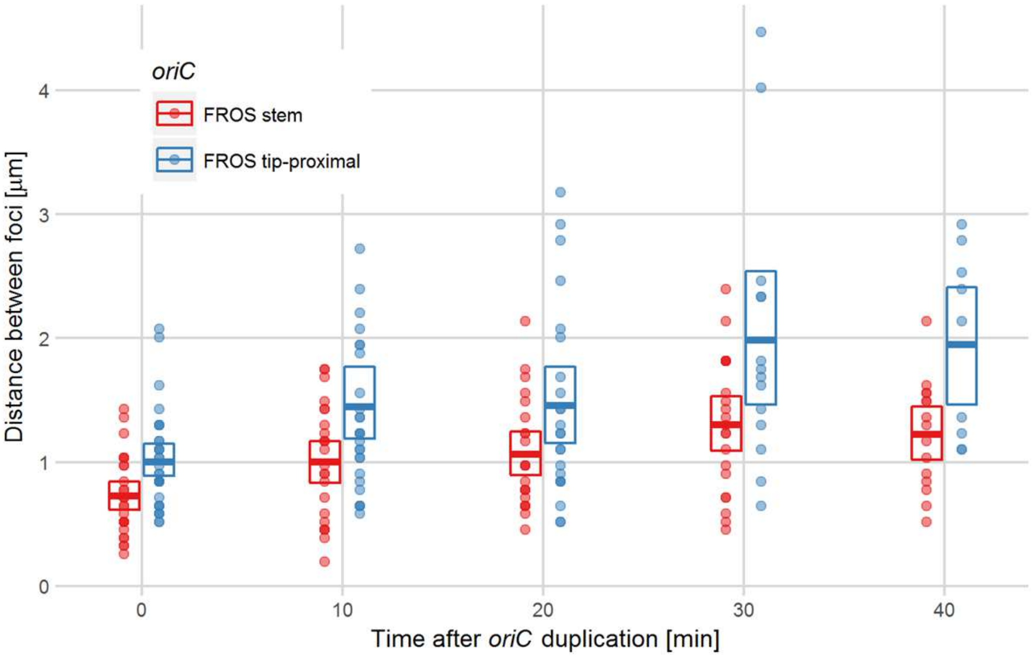

Fig. S10 Distance between tip-proximal and stem (tip-distal) FROS at the indicated time after their duplication in “wild type” FROS *dnaN-egfp* (AK122). Crossbars show the mean with 95% confidence intervals. The analysis was performed 27 stem and 32 tip-proximal FROS complexes.
