# Supplementary material for: Unique Function of the Bacterial Chromosome Segregation Machinery in Apically Growing Streptomyces - Targeting the Chromosome to New Hyphal Tubes and its Anchorage at the Tips
Source: PLoS Genet. 2016 Dec 15;12(12):e1006488. doi: 10.1371/journal.pgen.1006488 (PMC5157956; doi:10.1371/journal.pgen.1006488)
Supplement: S11 Fig — The images show separate channels: TetR-mCherry fluorescence (red) in the hyphae outlined and DIC images (grey), scale bar—1 μm. (PDF) [file pgen.1006488.s011.pdf]

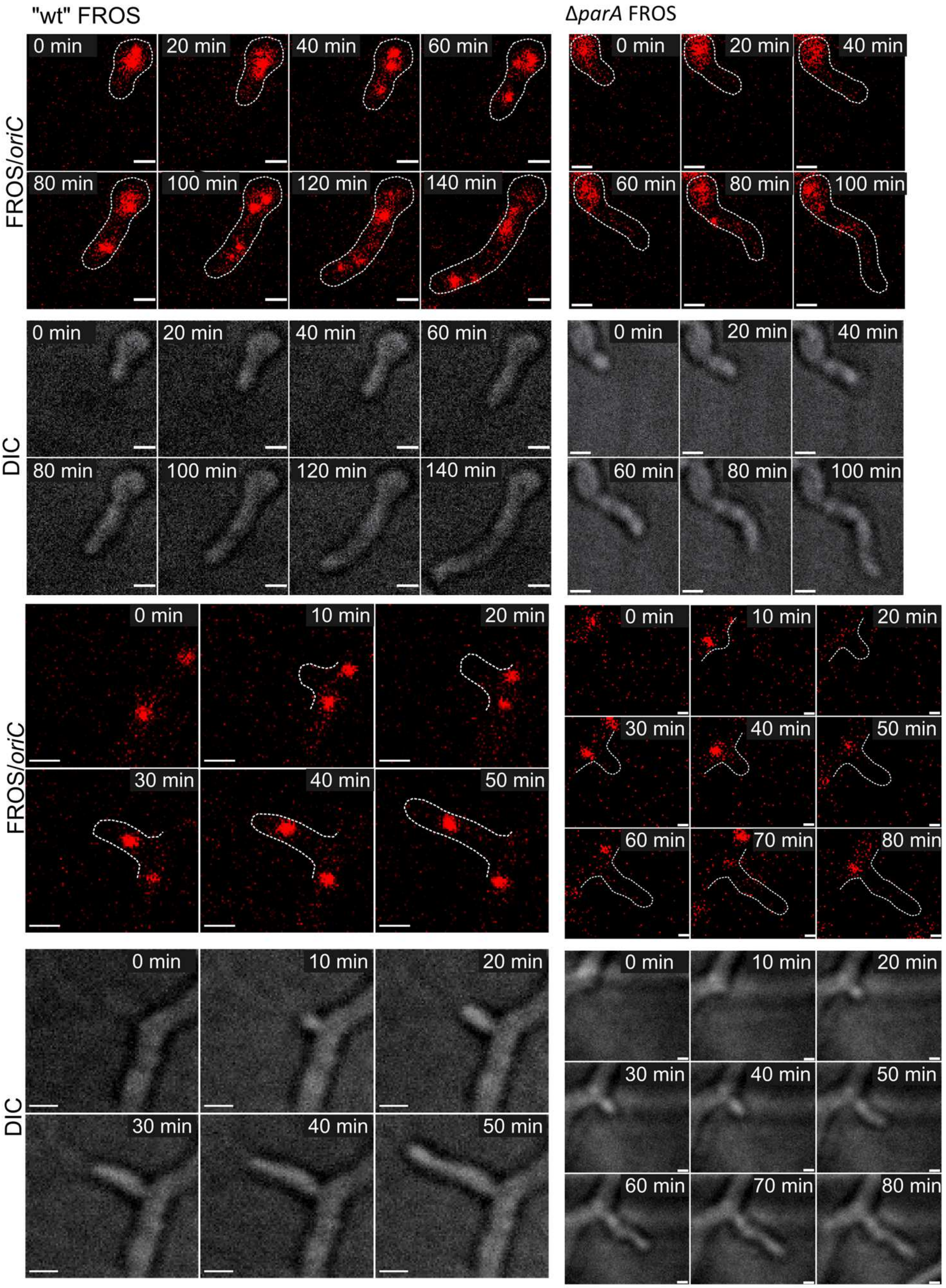


**Fig. S11 Time-lapse snapshots of FROS (TetR-mCherry fluorescence, red) in the germinating spores (top panel) and branching hyphae (bottom panel) of "wild type" FROS (DJ-NL102) and Δ*parA* FROS (AK115) strains.** The images show separate channels: TetR-mCherry fluorescence (red) in the hyphae outlined and DIC images (grey), scale bar - 1 μm.
