# Supplementary material for: Unique Function of the Bacterial Chromosome Segregation Machinery in Apically Growing Streptomyces - Targeting the Chromosome to New Hyphal Tubes and its Anchorage at the Tips
Source: PLoS Genet. 2016 Dec 15;12(12):e1006488. doi: 10.1371/journal.pgen.1006488 (PMC5157956; doi:10.1371/journal.pgen.1006488)
Supplement: S1 Table — (PDF) [file pgen.1006488.s017.pdf]

TABLE S1. Strains and plasmids used in this study

**Strains**	**Relevant genotype and characteristics**	**Source**
E. coli		
DH5α	*supeE44*, *ΔlacU169* (*ϕ80lacZΔM15*), *hsdr*17 (r_K-, m_K-), *recA1*, *endA1*, *gyrA96*, *thi-1*, *relA1*	Laboratory Stock
JM109	endA1 glnV44 thi-1 relA1 gyrA96 recA1 mcrB$^+$ Δ(lac-proAB) e14- [F' traD36 proAB$^+$ lacI^q lacZ ΔM15] sdR17($r_K^- m_K^+$)	[1].
BW25113 /pIJ790	K12 derivative: Δ*araBAD*, Δ*rhaBAD* /λ-Red (*gam, bet, exo*), *araC*, *rep101* (Ts), *cat^R*	[2]
ET12567 /pUZ8002	*dam*-13::Tn9, *dcm*, *hsdM*, *hsdR*, *zjj*-201::Tn10/*tra*, RP4, *cat^R*, *neo^R*	[3]
S. coelicolor		
M145	SCP1$^-$, SCP2$^-$	[4]
J3310	M145*parB-egfp*	[5]
J3305	M145Δ*parB*	[6]
J3306	M145Δ*parA*	[7]
J3318	M145*parB-egfp*Δ*parA*	[7]
J3337	M145*dnaN-egfp::apra* (apra^R)	[8]
J3337a	M145*dnaN-egfp scar* (apra^S)	This work
J3336	M145*dnaN-egfp::apra*Δ*parA*	This work
J3336a	M145*dnaN-egfp scar* Δ*parA* (apra^S)	This work
K112	M145Δ*scy*	[9]
BD05	M145Δ*scy, parB-egfp-apra*	This work
BD08	M145*parA S249Y, E250V*	[10]
DJ590	M145*parA-egfp*	[10]
DJ598	M145*parA S249Y, E250V parB-egfp-apra*	This work
DJ532	J3310 pIJ6902*parA*(hyg^R)	This work
EJTH31	M145 Tn5431*tetO-apra* (apra^R)	This work
DJ-NL102	M145 Tn5431*tetO-apra* pMS83-*mCherry* (apra^R, hyg^R)	This work
AK101	M145 *parB-egfp dnaN-mcherry::apra*	This work
AK113	J3310 Tn5431*tetO-apra,* pMS83-*mCherry* (apra^R, hyg^R)	This work
AK114	J3305 Tn5431*tetO-apra,* pMS83-*mCherry* (apra^R, hyg^R)	This work
AK115	J3306 Tn5431*tetO-apra,* pMS83-*mCherry* (apra^R, hyg^R)	This work
AK122	J3337a Tn5431*tetO-apra,* pMS83-*mCherry* (apra^R, hyg^R)	This work
AK123	J3336a Tn5431*tetO-apra,* pMS83-*mCherry* (apra^R, hyg^R)	This work
**Constructs**		
StH18	Supercos-1 containing chromosomal DNA from *S. coelicolor*; amp^R km^R, cosmid carrying *oriC* region	[11]
pIJ6902*parA* (hyg)	hyg^R pIJ6902-derivative, carrying *parA* under the control of the thiostrepton inducible *PtipA* promotor	[12]
pMS83	pMS81 with fragment containing *tetR* from pPC49 cloned in NsiI/KpnI sites, integrates at the $attB_{\varphi BT1}$ attachment site on the chromosome of *Streptomyces coelicolor*	[13]
pMS83-*mCherry*	*tetR-mCherry* under the control constitutive promoter from phage II9 SF14 in the intergrative plasmid	This work
pLAU44	Tandem *tetO* array	[14]
pIJ773	apra^R, *oriT*	[2]
pMOD<MCS>	Tn*5* mosaic ends	Epicentre

pUC44	*tetO* array cloned into pUC18	This work
p44FRT	$apra^R$, *oriT* cloned into pUC44	This work
p44FMOD	Source plasmid for Tn*5431*	This work
EJTH31A *(tetO)*	StH18 containing Tn*5431*, $apra^R$ and *oriT*	This work
StH18*dnaN-egfp*	Supercos-1 containing chromosomal DNA from *S. coelicolor*; amp^R km^R, cosmid carrying *egfp* downstream of *dnaN* gene	This work

1. Yanisch-Perron C, Vieira J, Messing J. Improved M13 phage cloning vectors and host strains: nucleotide sequences of the M13mp18 and pUC19 vectors. Gene. 1985;33: 103–119.
2. Gust B, Challis GL, Fowler K, Kieser T, Chater KF. PCR-targeted Streptomyces gene replacement identifies a protein domain needed for biosynthesis of the sesquiterpene soil odor geosmin. Proceedings of the National Academy of Sciences. 2003;100: 1541–1546.
3. Paget MS, Chamberlin L, Atrih A, Foster SJ, Buttner MJ. Evidence that the extracytoplasmic function sigma factor sigma E is required for normal cell wall structure in Streptomyces coelicolor A3(2). Journal of bacteriology. 1999;181: 204–211.
4. Kieser T, Bibb MJ, Buttner MJ, Chater KF, Hopwood DA. Practical Streptomyces Genetics. John Innes Centre Ltd. 2000; 529. doi:10.4016/28481.01
5. Jakimowicz D, Gust B, Zakrzewska-Czerwińska J, Chater KF. Developmental-Stage-Specific Assembly of ParB Complexes in Streptomyces coelicolor Hyphae. Journal of Bacteriology. 2005;187: 3572–3580. doi:10.1128/JB.187.10.3572
6. Kois A, Swiatek M, Jakimowicz D, Zakrzewska-Czerwińska J. SMC protein-dependent chromosome condensation during aerial hyphal development in Streptomyces. Journal of bacteriology. 2009;191: 310–9. doi:10.1128/JB.00513-08
7. Jakimowicz D, Zydek P, Kois A, Zakrzewska-Czerwińska J, Chater KF. Alignment of multiple chromosomes along helical ParA scaffolding in sporulating Streptomyces hyphae. Molecular microbiology. 2007;65: 625–41. doi:10.1111/j.1365-2958.2007.05815.x
8. Ruban-Ośmiałowska B, Jakimowicz D, Smulczyk-Krawczyszyn A, Chater KF, Zakrzewska-Czerwińska J. Replisome localization in vegetative and aerial hyphae of Streptomyces coelicolor. Journal of bacteriology. 2006;188: 7311–6. doi:10.1128/JB.00940-06
9. Holmes NA, Walshaw J, Leggett RM, Thibessard A, Dalton KA, Gillespie MD, et al. Coiled-coil protein Scy is a key component of a multiprotein assembly controlling polarized growth in Streptomyces. Proceedings of the National Academy of Sciences of the United States of America. 2013;110: E397-406. doi:10.1073/pnas.1210657110
10. Ditkowski B, Holmes N, Rydzak J, Donczew M, Bezulska M, Ginda K, et al. Dynamic interplay of ParA with the polarity protein, Scy, coordinates the growth with chromosome segregation in Streptomyces coelicolor. Open biology. 2013;3: 130006. doi:10.1098/rsob.130006
11. Redenbach M, Kieser HM, Denapaite D, Eichner A, Cullum J, Kinashi H, et al. A set of ordered cosmids and a detailed genetic and physical map for the 8 Mb Streptomyces coelicolor A3(2) chromosome. Mol Microbiol. 1996;21: 77–96.
12. Ditkowski B, Troć P, Ginda K, Donczew M, Chater KF, Zakrzewska-Czerwińska J, et al. The actinobacterial signature protein ParJ (SCO1662) regulates ParA polymerization and affects chromosome segregation and cell division during Streptomyces sporulation. Molecular microbiology. 2010;78: 1403–15. doi:10.1111/j.1365-2958.2010.07409.x
13. Gregory MA, Till R, Smith MCM. Integration Site for Streptomyces Phage ϕBT1 and Development of Site-Specific Integrating Vectors. Journal of bacteriology. 2003;185: 5320–5323. doi:10.1128/JB.185.17.5320
14. Lau IF, Filipe SR, Søballe B, Økstad O, Barre F, Sherratt DJ. Spatial and temporal organization of replicating Escherichia coli chromosomes. Molecular Microbiology. 2003;49: 731–743. doi:10.1046/j.1365-2958.2003.03640.x
