# Supplementary material for: Unique Function of the Bacterial Chromosome Segregation Machinery in Apically Growing Streptomyces - Targeting the Chromosome to New Hyphal Tubes and its Anchorage at the Tips
Source: PLoS Genet. 2016 Dec 15;12(12):e1006488. doi: 10.1371/journal.pgen.1006488 (PMC5157956; doi:10.1371/journal.pgen.1006488)
Supplement: S2 Table — (PDF) [file pgen.1006488.s018.pdf]

TABLE S2. Oligonucleotides used in this study

**Name**	**Sequence**	**Application**
cherry_f	CATATGTGGCCTGGAGAAACAGCTAAAGTGCGAAAGCGGCCTGC CGGGCCCGGAGCTGCCGGGCCCGGAGGTGAGCAAGGGCGAGG AGG	*mCherry* gene amplification
cherry_r	ATGCATTTACTTGTACAGCTCGTCCATGCC	
EZR1	ATGCGCTCCATCAAGAAGAG	Tn5431 sequencing [1]
DnaNspr_fw	GACGGTCTGAGCGCCATCGACT	verification of *dnaN* gene
DnaNspr_rv	GCGTCGAGCCTAAACCCGCAC	
ParBgfplink	CCTTCTGGAGGGCGAGGACGAGGACGGGGACGCCGAGTCCCTC C CGGGCCCGGAGCTG	*egfp-apra* amplification
Bdownrev	GGCCTTGTGCCAGGCGGCTTTGCTTT	
DnaNRSwa	GGACAGCCGCCGACGGCGCCACCGGCCGGCGGGCGACGGTATT TAAATTGTAGGCTGGAGCTGCTTC	mCherry-apra cassette amplification
DnaNF	GGCCTACAAGTACCTGATCATGCCGGTGCGGCTGAGCGGCCTGC CGGGCCCGGAGCTG	
Blap1apraRV	AATCTAAAGTATATATGAGTAAACTTGGTCTGACAGTTAAGTTCC CGCCAGCCTCGCAGA	*amp*^{R} gene replacement
Blap2apraFW	CCCTGATAAATGCTTCAATAATATTGAAAAAGGAAGAGTATAACT TATGAGCTCAGCCAATC	

1. Bishop A, Fielding S, Dyson P, Herron P. Systematic insertional mutagenesis of a streptomycete genome: a link between osmoadaptation and antibiotic production. Genome Research. 2004;14: 893–900. doi:10.1101/gr.1710304
