# Supplementary material for: Unique Function of the Bacterial Chromosome Segregation Machinery in Apically Growing Streptomyces - Targeting the Chromosome to New Hyphal Tubes and its Anchorage at the Tips
Source: PLoS Genet. 2016 Dec 15;12(12):e1006488. doi: 10.1371/journal.pgen.1006488 (PMC5157956; doi:10.1371/journal.pgen.1006488)
Supplement: S1 Text — (PDF) [file pgen.1006488.s019.pdf]

Supplementary Materials and Methods

Details of the strains construction

To construct the *S. coelicolor* strain carrying the *tetO* array, first we constructed a Tn*5* derivative, Tn*5431*, containing 120 tandemly arrayed copies of *tetO*, an apramycin resistance gene and an *oriT* site to facilitate delivery of the transposon to *S. coelicolor* following *in vitro* transposition to *S. coelicolor* cosmids [1,2]. To construct Tn*5431*, a 4400 bp fragment containing 120 *tetO* cassettes was excised from pLAU44 [3], a kind gift from Prof. David Sherratt, with EcoRI/BglII and ligated into the EcoRI/BamHI sites of pUC18, creating pUC44. The *apra-oriT* cassette was excised from pIJ773 [4] using EcoRI/HindIII and ligated into the same sites of pUC44 to create p44FRT. This plasmid was digested with NheI/HindIII to excise the *tetO-apra-oriT* fragment that was ligated into pMOD<MCS> digested with XbaI/HindIII. This created p44FMOD that carried the transposon Tn*5431* that was excised from p44FMOD using PvuII and used for *in vitro* transposition [1]. Cosmid StH18 [2,5], encompassing the *oriC* region (position 4269844-4270777 of the *S. coelicolor* chromosome) was selected for tagging with Tn*5431* by *in vitro* transposition [1]. Transposed cosmids were recovered by transformation of *E. coli* JM109 (selecting for apramycin and kanamycin resistance). Tn*5431* insertion sites were determined by DNA sequencing of transposed cosmids using sequencing primer EZR1 [1]. One transposed cosmid, termed EJTH31A, carried a copy of Tn*5341* integrated ~29 kb from *oriC* region (position 4240808 of the *S. coelicolor* genome; this position refers to the first base of the duplicated CCGTAGAGC Tn*5* target site), in the intergenic region between SCO*3855* (possible membrane protein) and SCO*3856* (probable peptidyl-prolyl cis-trans isomerase). The insertion was 956 bp upstream start codon of SCO*3855* and 575 downstream stop codon of SCO*3856,* and was expected not to interfere with neighbouring genes. This cosmid was selected for construction of an *oriC* tagged strain of *S. coelicolor* by intergeneric conjugation [6]. Analysis of transconjugant colonies showed that about 10% had the apra^R, km^S, phenotype that was indicative integration of Tn*5431* into the M145 chromosome via a double recombination event (S1A Fig). One of the transconjugants, termed EJTH31, was used for the subsequent introduction of *tetR-mcherry*.

To construct the *tetR-mcherry* fusion the *mCherry* gene was amplified from pRSET*cherry* (a kind gift from Prof. Roger Tsien) with primers cherry_f and cherry_r (S2 Table) and cloned between the NdeI and NsiI sites of pMS83 containing the *tetR* gene under the control of constitutive promoter from phage II9 SF14 [7]. EJTH31 was used to integrate pMS83-mCherry plasmid (in $att_{\varphi BT1}$ site) to yield the strain DJ-NL102 (FROS strain).

The cosmid EJTH31A and integrating plasmid pMS83-*mCherry* were introduced into *S. coelicolor* J3310 (*parB-egfp*)[8]; *S. coelicolor* J3305 (Δ*parB*)[9], *S. coelicolor* J3306 (Δ*parA*)[10], *S. coelicolor* J3337a (*DnaN-egfp*) and *S. coelicolor* J3336a (*DnaN-egfpΔparA*) (see below). Apra^R, km^S,

hyg^R exconjugates were selected indicating a double recombination of Tn*5431* and integration of pMS83-mCherry. The obtained strains were verified by PCR and Southern hybridisation. Additionally, SDS-PAGE analysis confirmed the production of the TetR-mCherry fusion protein (S1 Fig).

To introduce Tn*5431* to *S. coelicolor* expressing *dnaN-egfp* we modified J3337 [11] and J3336 (constructed by recombination of J3306 (Δ*parA*) strain with H18 *dnaN-egfp-apra* cosmid), to remove the apramycin resistance cassette, yielding strains J3337a and J3336a. The apramycin cassette was removed from the cosmid H18 *dnaN-egfp-apra* [11] by using FLP recombinase to yield StH18*dnaN-egfp*. Subsequently the *amp* gene in SuperCos of StH18*dnaN-egfp* was exchanged for *apra-oriT* cassette (amplified from pIJ773 with primers Blap1apraFW and Blap1apraRV). This construct was used for conjugation into *S. coelicolor* J3337 and J3336. Exconjugants were screened for the loss of both apra^R and Km^R, indicating a double cross-over allelic exchange of *dnaN* locus. The obtained strains J3337a and J3336a were verified by PCR using primers DnaNspr_fw and DnaNspr_rv (S2 Table). The presence of DnaN-EGFP in *S. coelicolor* cell extracts was examined by scanning SDS-PAGE gel with a scanner equipped with 488-nm laser.

In order to construct DJ598 strain ($parA_{mut}$, *parB-egfp*), *egfp–apra* cassette was amplified using primers ParBgfplink and Bdownrev, as described before (S2 Table) [8] and was used to target the cosmid H24*parA S249Y,E250V* [12] downstream of *parB* gene. The cosmid H24*parA S249Y,E250VparB-egfp-apra* was used for conjugation into *S. coelicolor* strain BD08 [12]. Apra^R exconjugants were screened for the loss of km^R, indicating the presence of a double cross-over allelic exchange, yielding DJ598 strain (S1 Table).

To obtain the strain overproducing ParA (DJ532) we used the construct pIJ6902*parA* in which *parA* was expressed from the thiostrepton-inducible *tipA* promoter [13]. The pIJ6902*parA(hyg)* plasmid was introduced into *S. coelicolor* J3310 (*parB-egfp*) [8], yielding DJ532 (S1 Table). The increased level of ParA (about 4 times exceeding wild type level) for cultures grown in presence of thiostrepton (5µg/ml) was confirmed by Western blot with antibodies against ParA.

Strain BD05 was obtained by recombination of K112 [14] with H24 *parB-egfp-apra* cosmid and apra^R exconjugants were screened for the loss of km^R, indicating the presence of a double cross-over allelic exchange.

AK101 strain was constructed by recombination of J3310 strain (*parB-egfp*) with H18 *dnaN-mcherry-apra* cosmid. First the *mcherry-apra* cassette amplified with primers DnaNRSwa and DnaNF and recombined with H18 using PCR targeting. H18 *dnaN-mcherry-apra* cosmid was introduced to J3310 strain and apra^R exconjugants were screened for the loss of km^R, indicating the presence of a double cross-over allelic exchange. The presence of DnaN-mCherry in *S. coelicolor* cell extracts was examined by scanning SDS-PAGE gel with a scanner equipped with 488-nm laser.

References

1. Bishop A, Fielding S, Dyson P, Herron P. Systematic insertional mutagenesis of a streptomycete genome: a link between osmoadaptation and antibiotic production. Genome Research. 2004;14: 893–900. doi:10.1101/gr.1710304
2. Redenbach M, Kieser HM, Denapaite D, Eichner A, Cullum J, Kinashi H, et al. A set of ordered cosmids and a detailed genetic and physical map for the 8 Mb Streptomyces coelicolor A3(2) chromosome. Mol Microbiol. 1996;21: 77–96.
3. Lau IF, Filipe SR, Søballe B, Økstad O, Barre F, Sherratt DJ. Spatial and temporal organization of replicating Escherichia coli chromosomes. Molecular Microbiology. 2003;49: 731–743. doi:10.1046/j.1365-2958.2003.03640.x
4. Gust B, Challis GL, Fowler K, Kieser T, Chater KF. PCR-targeted Streptomyces gene replacement identifies a protein domain needed for biosynthesis of the sesquiterpene soil odor geosmin. Proceedings of the National Academy of Sciences. 2003;100: 1541–1546.
5. Bentley SD, Chater KF, Cerdeño-Tárraga, AM Challis G, Thomson N, James K, Harris D, et al. Complete genome sequence of the model actinomycete Streptomyces coelicolor A3(2). Nature. 2004;417(: 141–7.
6. Kieser T, Bibb MJ, Buttner MJ, Chater KF, Hopwood DA. Practical Streptomyces Genetics. John Innes Centre Ltd. 2000; 529. doi:10.4016/28481.01
7. Gregory MA, Till R, Smith MCM. Integration Site for Streptomyces Phage ϕ BT1 and Development of Site-Specific Integrating Vectors. Journal of bacteriology. 2003;185: 5320–5323. doi:10.1128/JB.185.17.5320
8. Jakimowicz D, Gust B, Zakrzewska-Czerwińska J, Chater KF. Developmental-Stage-Specific Assembly of ParB Complexes in Streptomyces coelicolor Hyphae. Journal of Bacteriology. 2005;187: 3572–3580. doi:10.1128/JB.187.10.3572
9. Kois A, Swiatek M, Jakimowicz D, Zakrzewska-Czerwińska J. SMC protein-dependent chromosome condensation during aerial hyphal development in Streptomyces. Journal of bacteriology. 2009;191: 310–9. doi:10.1128/JB.00513-08
10. Jakimowicz D, Zydek P, Kois A, Zakrzewska-Czerwińska J, Chater KF. Alignment of multiple chromosomes along helical ParA scaffolding in sporulating Streptomyces hyphae. Molecular microbiology. 2007;65: 625–41. doi:10.1111/j.1365-2958.2007.05815.x
11. Ruban-Ośmiałowska B, Jakimowicz D, Smulczyk-Krawczyszyn A, Chater KF, Zakrzewska-Czerwińska J. Replisome localization in vegetative and aerial hyphae of Streptomyces coelicolor. Journal of bacteriology. 2006;188: 7311–6. doi:10.1128/JB.00940-06
12. Ditkowski B, Holmes N, Rydzak J, Donczew M, Bezulska M, Ginda K, et al. Dynamic interplay of ParA with the polarity protein, Scy, coordinates the growth with chromosome segregation in

Streptomyces coelicolor. Open biology. 2013;3: 130006. doi:10.1098/rsob.130006
13. Ditkowski B, Troć P, Ginda K, Donczew M, Chater KF, Zakrzewska-Czerwińska J, et al. The actinobacterial signature protein ParJ (SCO1662) regulates ParA polymerization and affects chromosome segregation and cell division during Streptomyces sporulation. Molecular microbiology. 2010;78: 1403–15. doi:10.1111/j.1365-2958.2010.07409.x
14. Holmes NA, Walshaw J, Leggett RM, Thibessard A, Dalton KA, Gillespie MD, et al. Coiled-coil protein Scy is a key component of a multiprotein assembly controlling polarized growth in Streptomyces. Proceedings of the National Academy of Sciences of the United States of America. 2013;110: E397-406. doi:10.1073/pnas.1210657110
